# Hybrid CNN–Transformer Framework for Automated Detection of Developmental Coordination Disorder from Motion Imaging Sequences

**DOI:** 10.3390/bioengineering13090970

**Published:** 2026-08-25

**Authors:** Khaled Mahmoud Heba, Abbas Hassan Abbas Atya, Noor Hazim Saleh Alrawashdeh, Sana Shahab, Mohd Anjum

**Affiliations:** 1 Department of Architecture, College of Engineering and Information Technology, Onaizah Colleges, Onaizah 56447, Saudi Arabia; 2 King Salman Center for Disability Research, Riyadh 11614, Saudi Arabia; 3 Department of Architecture, Faculty of Engineering, Al-Azhar University, Nasr City, Cairo 11884, Egypt; 4 Department of Electrical Engineering, College of Engineering and Information Technology, Onaizah Colleges, Onaizah 56447, Saudi Arabia; 5 Karak Government Hospital (KGH), Al Karak 61101, Jordan; 6 Department of Business Administration, College of Business Administration, Princess Nourah Bint Abdulrahman University, Riyadh 11671, Saudi Arabia; 7 Department of Computer Engineering, Aligarh Muslim University, Aligarh 202002, India

**Keywords:** developmental coordination disorder, hybrid CNN–transformer, spatiotemporal feature learning, optical flow maps, pose estimation skeletons, cross-modal fusion, motion imaging sequences, neurodevelopmental diagnostics

## Abstract

Hybrid CNN–Transformer (HCT) synthesis for automated neurodevelopmental diagnostics is an effective approach to constructing intelligent detection systems that are not merely oriented toward feature classification but primarily toward solving spatiotemporal pattern recognition problems in motor disorder assessment. In neurodevelopmental diagnostics, existing automated methods rely on fixed, single-model architectures that process spatial or temporal motion features independently, failing to adapt to the heterogeneous motor irregularities characteristic of developmental coordination disorder and degrading detection sensitivity and generalization across diverse patient populations. There is therefore a pressing need for models capable of simultaneously capturing intra-frame spatial coordination patterns and inter-frame temporal movement dependencies against interrelated diagnostic criteria including accuracy, sensitivity, and motor irregularity specificity. To address this challenge, this paper proposes HCT, a novel framework that integrates ResNet-based spatial feature extraction from optical flow maps and pose estimation skeletons with multi-head self-attention Transformer encoding for modeling long-range temporal dependencies across multi-frame motion sequences. Unlike conventional single-stream approaches, where spatial and temporal processing remain confined to independent architectures, HCT decouples spatiotemporal feature learning through a cross-modal fusion pipeline, constructing a unified discriminative architecture that captures motor coordination dependencies between motion imaging inputs and multiple diagnostic criteria simultaneously. The convolutional encoder generates diverse joint displacement features, which are consolidated through cross-modal attention fusion into a robust, unified embedding with enhanced generalization and resilience to inter-individual motor variability. Integration within neurodevelopmental assessment frameworks facilitates reliable developmental coordination disorder classification, motor irregularity prediction, and interpretable diagnostic decision support, advancing the accuracy, flexibility, and clinical validity of intelligent motor disorder diagnostic systems.

## 1. Introduction

Motor coordination neurodevelopmental disorders have recently become a major public health challenge because of their long-term effects on children’s academic performance, social participation, cognitive development, and psychological well-being [1]. Developmental Coordination Disorder (DCD) is one of the most common but least diagnosed subgroups of childhood motor problems, characterized by persistently poor coordination, difficulty with balance control and postural regulation, and difficulty with the execution of age-appropriate motor tasks [2]. Children with DCD often exhibit atypical gait synchrony, significantly delayed hand–eye coordination, late motor alterations such as fine motor skills and body balancing, and movement sequence disorders [3,4], severely affecting their activities of daily living like handwriting, dressing, and object handling, and transportation activities such as locomotion or even physical education-related skills [5]. While it is highly prevalent across pediatric populations, early identification of DCD remains a clinical conundrum, as motor abnormalities present in a heterogeneous manner that overlaps with symptoms specific to other neurodevelopmental conditions such as autism spectrum disorder, attention-deficit hyperactivity disorder, and cerebral motor dysfunction [6].

Diagnosis of this disorder has largely relied on clinical observation, standardized motor assessment protocols, caregiver questionnaires, and expert interpretation of performance metrics [7]. These approaches have clinical utility but are inherently subjective and subject to inter-rater variability, and exhibit inconsistent reproducibility; evaluation procedures are resource-intensive and non-scalable in many pediatric populations. Furthermore, many approaches derived from classical diagnostics analyze partial motion patterns without fully integrating the temporal continuity and spatial dependencies inherent to higher-order movement sequences [8]. These limitations are especially problematic when analyzing subtle motor coordination abnormalities, particularly in the clinical setting, where developmental stages are rather non-specific. As a matter of fact, such methodological limitations significantly limit sensitivity, rendering the diagnostic inference less clinically relevant. The result is an increasing focus of research on automated intelligent diagnostic systems that use motion imaging data to extract discriminative spatiotemporal movement representations.

The world of neurodevelopmental research has seen tremendous progress and breakthroughs in the development of automated evaluation tools, alongside periodic reassessment and intervention [9]. Given advances in computer vision, deep learning, motion analysis technologies, automated systems based on video motion capture, optical flow analysis techniques, and skeletal pose estimation methods across different imaging modalities, we are beginning to see significant strides toward scalable clinical implementation of this approach. Convolutional Neural Networks (CNNs) capture spatial representations from sequences of video and imaging frames; thus, early deep learning methods for motor disorder detection primarily leverage CNNs as backbones in the absence of relevant global features [10]. The designed CNN architectures proved to fully utilize local texture information, joint displacement patterns, posture irregularities, and motion gradients from imaging data. By design, traditional CNN-based networks place strong emphasis on local receptive fields and short-range feature dependencies, limiting their ability to capture long-term temporal relationships that maintain the continuity of joint coordination across successive movement frames [11]. Motor impairments in DCD physiology often manifest as temporal patterns, including successively coordinated limb movements, compensatory repetition of balancing movements, and compounded inconsistencies in motion that cannot be resolved by extracting isolated spatial features.

To address the issue of modeling temporal dependencies, architectures operating on sequential movement data were developed: recurrent models such as Long Short-Term Memory [12] networks and Gated Recurrent Units [13]. These models advanced the ability of temporal learning by retaining historical information on movements over multiple video data points. However, recurrent frameworks have intrinsic limitations: instability of gradients; limited parallelization due to sequential processing; struggles with long-range dependency representation; and reduced robustness to high-dimensional motion sequences containing diverse motor faults. Moreover, current techniques typically follow single-stream processing pipelines that ensure that either spatial imaging features or temporal skeletal dynamics independently dominate the learning process, thus limiting the thorough integration of multimodal movement information necessary for accurate characterization of DCD.

Transformer-based architectures [14] with multi-head self-attention mechanisms have achieved state-of-the-art performance in modeling long-range contextual relationships across a wide range of sequential data domains, including natural language processing, medical imaging, human activity recognition, and more. Self-attention mechanisms can capture global dependencies between temporally distant movement representations without recursively propagating through structural components, thereby modeling complex motor coordination trajectories more effectively. Yet Transformer models typically require large-scale training datasets and often struggle to learn local spatial representations when directly applied to medical motion imaging tasks. Over-reliance on lone Transformer architectures is particularly limiting for neurodevelopmental diagnostics, where clinically annotated datasets are scarce, and movement abnormalities are highly nuanced; this can lead to poor generalization stability and limited extraction of discriminative features.

As a result, the combination of CNN and Transformer architectures [15] has become an attractive approach for designing spatiotemporal hybrid learning models that can learn local spatial information and global temporal dependencies simultaneously. Hybrid architectures enable complementary learning behavior, where convolutional layers can extract discriminative motion-appearance features while Transformer encoders summarize sequential coordination relationships over a larger temporal scale. This work refers to the conventional systems for assessing motor disorders that are powered by hybrid vision architectures, given recent progress in this area, and demonstrates that the methods still exhibit important but unresolved limitations. Many frameworks use naive feature concatenation approaches that lack adaptive cross-modal interaction between optical flow and skeletal pose dynamics. Importantly, many approaches are limited to activity recognition rather than to clinically interpretable neurodevelopmental diagnostics, given heterogeneous patient populations and diverse motor presentations. This paper presents a Hybrid CNN–Transformer (HCT), a novel intelligent automated framework for DCD detection from motion imaging sequences, to tackle these challenges. The system has a unified spatiotemporal learning architecture that incorporates a ResNet backbone with self-attention to perform convolutional feature extraction and temporal dependency encoding to construct an end-to-end learnable motor development assessment framework. First, dense optical flow maps and a sequence of pose estimation skeletons are computed from the input motion imaging fields to capture different aspects of body movements: relatively coarse overall dominant force dynamics, as well as surrounding spatial structures in terms of joint positions, reflecting more detailed physical articulations. The convolutional encoder learns discriminative spatial representations of joint displacement distributions, body posture variations, motion trajectory irregularities and inter-limb coordination patterns. Then, the multi-head self-attention transformer represents long-range temporal dependencies and governs sequential motor behavior across multi-frame motion samples.

Differently from conventional dual-stream architectures that process spatial and temporal features independently, the proposed HCT provides a cross-modal fusion strategy to adaptively integrate heterogeneous movement representations into a unified discriminative embedding space. The fusion mechanism increases robustness to inter-subject motor variability, motion execution inconsistencies, environment imaging variations, and partial skeleton estimation noise. The framework successfully encodes clinically interpretable diagnostic features by jointly modeling optical flow dynamics and skeletal coordination patterns, therefore capturing subtle motor irregularities associated with DCD.

The proposed Hybrid CNN–Transformer (HCT) framework introduces methodological novelty through the design of a domain-oriented spatiotemporal representation learning architecture for automated DCD detection rather than through the formulation of a completely new deep learning algorithm. The novelty is established by a hierarchical dual-stream learning paradigm that simultaneously exploits optical flow maps and pose-estimation skeletons as complementary motion representations, enabling the extraction of both appearance-driven motion dynamics and biomechanical joint movement characteristics. An adaptive cross-modal attention fusion mechanism performs context-aware feature interaction to model inter-modal dependencies and semantic consistency between the two representations, producing a unified discriminative embedding that preserves both local spatial coordination and long-range temporal movement relationships. This representation is subsequently refined by Transformer-based temporal reasoning to capture movement continuity and motor execution patterns associated with DCD. Unlike conventional Hybrid CNN–Transformer architectures developed for generic action recognition; the proposed framework is specifically designed to characterize clinically relevant motor coordination abnormalities through structured cross-modal representation learning.

According to the discussions, the main objectives of this work are listed as follows.

1. A novel HCT architecture is proposed to automatically detect DCD from motion imaging sequences, capable of extracting spatial coordination patterns and temporal movement dependencies in a computationally practicable time.
2. A multimodal motion representation strategy combined with optical flow maps and pose estimation skeleton sequences is introduced to facilitate the quantitative characterization of heterogeneous motor coordination abnormalities.
3. The convolutional encoder based on ResNet can extract discriminative local movement features of posture instability, irregular joint displacements, and inconsistencies in motion trajectories.
4. A multi-head self-attention Transformer encoder is incorporated to capture long-range temporal relationships underlying coordinated sequential motor behavior over long motion sequences.
5. To realize more robust, generalizable, and inter-individual movement-variability-resistant unified cross-modal shallow spatiotemporal embeddings from this modally balanced pseudo dataset, a cross-modal attention fusion module is devised.

The rest of the paper is organized as follows: Section 2 describes the various researchers’ opinions regarding the DCD diagnostic frameworks. Section 3 explains the working process of the HCT architecture, and Section 4 explains the diagnostic process and the framework’s efficiency. Conclusions are described in Section 5.

## 2. Related Works

### 2.1. CNN-Based Motor Disorder and Gait Analysis Frameworks

Since CNNs have demonstrated immense potential for learning discriminative spatial representations from motion imaging data, the method has been widely used for automated assessment of movement disorders. Recent work has shown that CNN-like architectures can detect posture irregularities, gait abnormalities, limb motion inconsistencies, and motion characteristics during rehabilitation. Shi et al. [16] proposed an MPL-CNN framework for effective detection of rehabilitation training in patients with upper-limb movement disorders, leveraging intelligent motion analysis techniques. Their model used multi-path learning structures to enhance the diversity of features extracted from upper limb movement sequences and thus increase the accuracy of rehabilitation evaluation. The study showed that a CNN-based framework can effectively capture localized movement coordination patterns and rehabilitation-related motor changes. However, the architecture focused mainly on spatial representation learning rather than sufficient modeling of long-range temporal coordination dependencies in the execution of motor behavior over time.

Erdaş and Sümer [17] proposed a CNN-based model to classify neurodegenerative diseases using gait data represented as QR codes. The author introduced their framework, which converted gait motion signals into a QR-based visual representation to enhance feature discrimination via convolutional learning. The proposed approach achieved higher accuracy in classifying gaits for neurodegenerative disorders, detecting posture and locomotion irregularities from gait image encodings. However, the system could only process the analysis of static information representation and was unable to learn temporal dependencies within longer movement sequences. Zhou et al. [18] proposed a lightweight CNN-based attention mechanism to establish a portable framework for a gait assessment system in post-stroke rehabilitation monitoring. They combined visual gait analysis and attention-guided convolutional learning to enhance computational efficiency and movement recognition accuracy in rehabilitation environments. The light CNN detected gait instability and postural imbalance under resource-limited conditions. Although the model offered efficiency benefits, it primarily addressed the dynamics of short-range movements. It did not include a full model of sequential coordination required for evaluating complex neurodevelopmental disorders like DCD. Ma et al. [19] proposed a temporal dependency-learning CNN with attention modules for motor imagery EEG decoding. Their architecture showed that attention can enhance the temporal feature sensitivity and the quality of the discriminative representation in the sequential analysis of neurological signals. Despite its ability to enhance the temporal dependency learning over traditional CNNs, the framework only models temporal dependencies within a small spatiotemporal receptive field, limiting its effectiveness for understanding long-range spatiotemporal coordination abnormalities in motion imaging sequences. Overall, these studies have demonstrated the usefulness of CNNs for extracting local motion features, gait irregularity, and posture abnormalities. Nevertheless, convolutional frameworks naturally favor learning spatial representations at local positions, without attention to temporal coordination across the time series of phases in sequential movements, where long-range dependencies often exist and are related to motor impairments in DCD, requiring their comprehensive modeling.

### 2.2. Temporal Dependency Learning and Transformer-Based Motion Modeling

However, the scope of local convolutional learning is limited, and recent work has begun to adopt Transformer networks and temporal dependency models for sequential movement analysis. The transformer-based framework uses a self-attention mechanism to capture global context for temporally distributed motion sequences. Gai et al. [20] proposed a spatiotemporal Transformer model for video-based human pose estimation. The author managed to model long-range spatial and temporal interactions among articulated body parts within a framework well suited to capturing attention-guided interactions among features. In coordinating the motion structures of the skeleton globally, the Transformer architecture demonstrated strong performance. However, the framework primarily addressed general pose estimation tasks and was not clinically oriented regarding mechanisms for detecting subtle motor deficits in the neurodevelopmental domain. To optimize human health assessment, Liang et al. [21] proposed a spatial–temporal Transformer architecture for skeleton-based sports action recognition. The neural network had a self-attention encoding mechanism to learn dynamic relationships between the skeleton joints and the temporal body movement trajectory. Experimental analysis showed that recognition of complex sports actions involving coordinated limb interactions was improved. Although it had strong temporal learning, the system was primarily designed to recognize activities rather than to detect motor abnormalities in a clinically interpretable manner. To review the advancement of spatiotemporal prediction methods, ranging from Transformer architectures to large-scale foundation models, Mao et al. [22] carried out a thorough survey. The transformer-based models excel at understanding long-range sequential relationships in complex spatiotemporal data. A key finding of the survey was that self-attention mechanisms enable better context adaptability than recurrent and convolutional approaches. The study also highlighted key issues with standalone Transformer models, such as computational complexity, reliance on large datasets, and limited local spatial inductive bias. In studying motor sequence learning, Kashefi et al. [23] showed that predicting the next motion step and adjusting the trajectory accordingly is crucial for optimization. Their results validated the sequential dependency modeling and temporal movement continuity as key building blocks of human motor coordination analysis. This observation further underscores the need for long-range temporal learning in automated DCD detection systems. Transformer-based systems are known to have strong global contextual learning, but also suffer from a lack of local spatial representation learning and require large amounts of annotated data for stable optimization. Therefore, combining convolutional feature extraction with the temporal encoding using Transformers becomes crucial for a comprehensive assessment of neurodevelopmental movements.

### 2.3. Multimodal Fusion and Cross-Modal Attention Learning

Multimodal learning strategies that combine heterogeneous movement representations, such as skeletal motion, optical flow dynamics, physiological signals, and patterns of behavioral imaging, are now commonly used in recent intelligent diagnostic systems. The integration of complementary learning from diverse diagnostic perspectives within a cross-modal fusion framework enables complementary representation learning. To recognize stereotyped movements in children with autism spectrum disorder, Zhang et al. [24] introduced a multi-channel fusion framework and an action pattern mining method. The designed architecture includes multiple motion-representation channels, thereby enhancing sensitivity to repetitive behavior patterns for improved motion recognition. Experimental results showed that multi-channel fusion greatly enhances the robustness against movement variability and environmental inconsistencies. The overall approach to the fusion strategy, however, was largely based on channel aggregation with minimal interaction and adaptation between modalities. Liu et al. [25] proposed a multi-scale cross-modal attention dynamic decision fusion network for quantitative grading of Parkinson’s disease. Their model was based on cross-modal attention learning that dynamically combines heterogeneous diagnostic representations at multiple scales. The adaptive fusion strategy could provide more accurate estimates of disease severity and improve robustness in complex neurological movement conditions. However, the focus was on grading of Parkinsonian symptoms instead of assessment of pediatric neurodevelopmental motor disorders.

Dong et al. [26] proposed a symmetric cross-modal EEG–EMG attentional fusion framework to recognize unilateral upper-limb fine-motor imagery. They used bidirectional cross-modal attention to build dynamic interactions between neural and muscular signal representations within their system. Experimental results have shown that symmetric attention fusion offers better fine-motor recognition sensitivity and movement pattern consistency. Although it has been successfully used for multimodal signal integration, the research did not account for motion-imaging sequence analysis. Zhou et al. [27] designed a framework for cross-modal disentanglement and attention-alignment learning to assess attention-emotion in children with autism spectrum disorder. Their model was based on adaptive representation alignment mechanisms to improve interpretability and emotion evaluation accuracy across different modalities. The study emphasized the significance of cross-modal interaction learning in the assessment system of pediatric neurodevelopment. Hameed et al. [28] proposed a temporal–spatial Transformer framework for classification of MIM based on ICI in a brain–computer interface application. The researchers introduced a model that combines Transformer-based self-attention learning and temporal signal decomposition to boost motor imagery classification accuracy. The study demonstrated the effectiveness of attention-guided temporal dependency modeling for sequentially represented biomedical motion data. The framework, however, was primarily designed to analyze EEG signals rather than to assess skeletal coordination and motion images. Inspired by this, Teng et al. [29] introduced a multimodal Parkinson’s disease diagnosis system, ModFus-PD, that combines cross-modal attention methods with contrastive learning. Their architecture showed that multimodal interaction with attention leads to better representation discriminability and greater disease classification reliability. The framework successfully modeled inter-modal relationships between the different modalities, with minimal inter-modality redundancy. The method was optimized, however, for adult diagnosis of neurodegenerative disease rather than motion-sequence-based DCD analysis. All of these studies show that cross-modal attention systems significantly enhance the robustness, adaptability, and discriminative representation learning in heterogeneous neurological and motor assessment settings. However, current multimodal fusion-based systems are primarily focused on processing neurological signals, autism spectrum disorder behavioral analysis, or Parkinson’s disease grading, rather than on assessing spatiotemporal movement coordination pertinent to DCD diagnosis.

Chen et al. [30] proposed LS-ViT, which enhances Vision Transformer by integrating long- and short-term temporal difference modeling to capture multi-scale motion dynamics for action recognition, demonstrating improved temporal feature discrimination. Qin et al. [31] introduced Video Swin-CLSTM Transformer, where optical flow information is fused with a Video Swin Transformer backbone and ConvLSTM modules to strengthen long-range temporal dependency learning while suppressing background interference, resulting in improved recognition accuracy for complex motion sequences. Rahaman et al. [32] employed TimeSformer for video understanding by utilizing factorized spatiotemporal self-attention to model global motion relationships across consecutive frames, illustrating the effectiveness of Transformer-based architectures for sequence-level action analysis. Shin et al. [33] developed a dual-stream deep learning framework for autism spectrum disorder detection using skeleton-based body movement analysis, demonstrating that complementary skeletal motion representations substantially improve neurodevelopmental disorder classification by preserving fine-grained body coordination patterns.

### 2.4. Research Gap and Motivation

Current motor disorder assessment systems have drawbacks, including the inability to model temporal dependencies over long time periods, poor integration of both spatial and temporal motor representations, and limited flexibility in handling variability in pediatric motor abnormalities. CNN-based approaches have demonstrated their capacity to learn local posture features, but the methods have not been able to model sequential coordination behavior; the same holds for Transformer-based systems, which cannot learn fine-grained spatial features. Furthermore, most multimodal frameworks have been developed to recognize general activity or adult neurological disorders, rather than a clinically interpretable DCD assessment. Inspired by these challenges, the proposed HCT framework comprises three components: spatial feature extraction via ResNet, temporal modeling via transformer, and the fusion of optical flow and skeletal pose representation via cross-modal attention. The monolithic architecture enhances spatiotemporal movement learning, diagnostic reliability, and automated classification of DCD in the context of intelligent neurodevelopmental assessment.

The proposed Hybrid CNN–Transformer (HCT) framework differs from existing CNN–Transformer hybrid architectures through the design of a cross-modal attention-guided spatiotemporal representation learning strategy that performs adaptive interaction between heterogeneous motion modalities instead of independent feature extraction followed by conventional fusion. Specifically, optical flow maps encode dense motion dynamics, whereas pose-estimation skeletons represent biomechanical joint configurations and movement trajectories. The proposed cross-modal attention mechanism establishes semantic correspondence between these complementary representations by dynamically assigning attention weights according to their contextual relevance, thereby reinforcing mutually informative features while suppressing redundant and inconsistent responses. Unlike existing multimodal fusion approaches based on feature concatenation, weighted averaging, or late-decision fusion, the proposed mechanism performs feature alignment and contextual refinement before Transformer-based temporal modeling, enabling joint optimization of spatial motion characteristics and long-range movement dependencies within a unified latent space. This hierarchical representation learning strategy captures clinically significant motor coordination patterns, inter-joint movement relationships, and temporal execution inconsistencies that are characteristic of DCD.

## 3. Hybrid CNN–Transformer Framework for Automated Detection of Developmental Coordination Disorder

**Figure 1:**
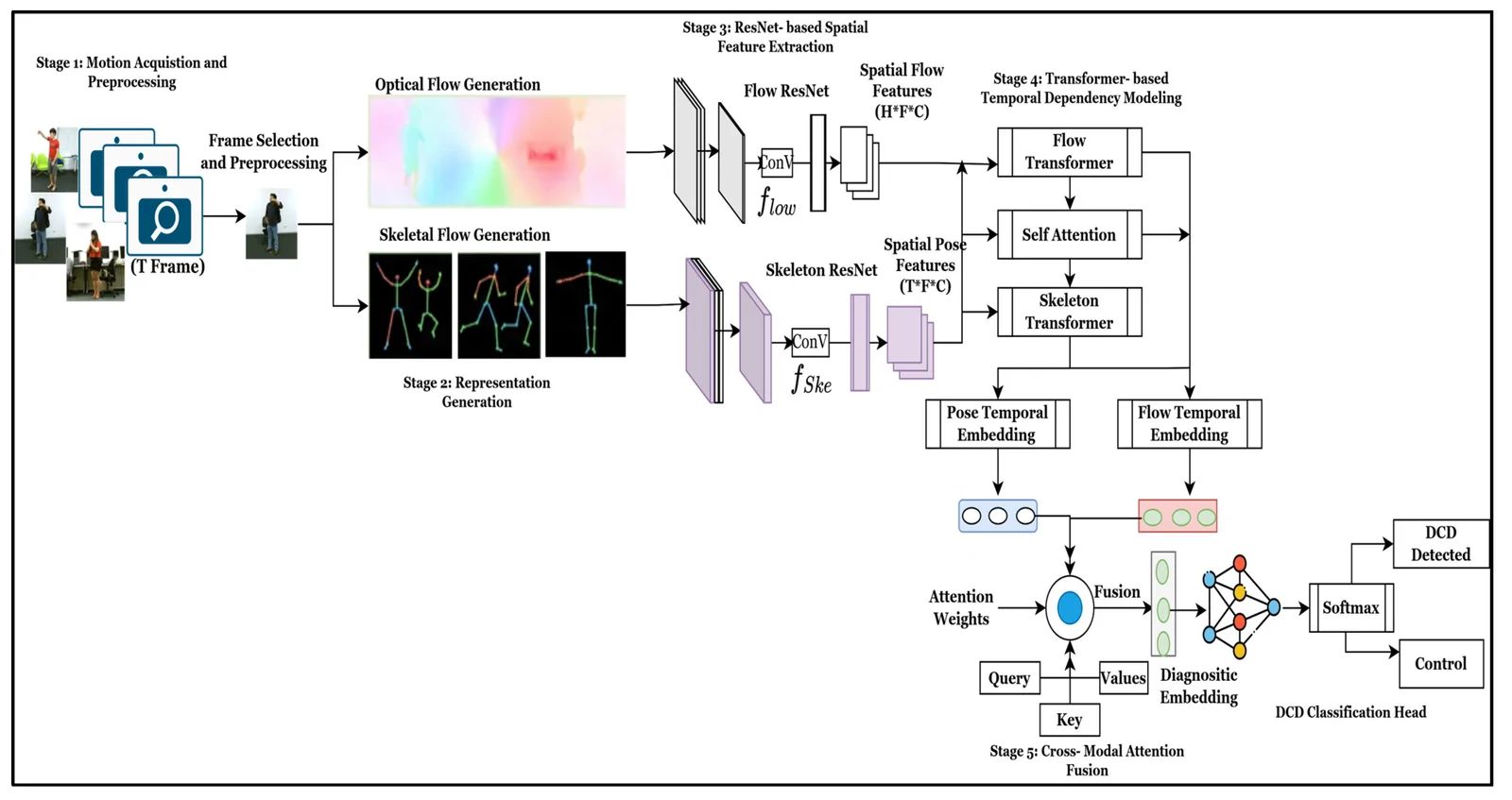
Overall architecture of Hybrid CNN–Transformer framework.

The proposed HCT framework aims to automate DCD detection by handling spatiotemporal information in a unified manner from motion imaging sequences. The framework receives input motion videos with *V* frames in time, and after performing pre-processing steps such as normalization, resizing, Gaussian filtering, and background subtraction, a set of representative frames S for N frames is created from the video. The processed sequences are used to generate two complementary representations of motion: optical flow maps $OF\in R^{N\times H\times W\times 2}$ that represent dense motion displacement in the H×W space, and skeletal pose representations $SK\in R^{N\times K\times 2}$, where K is the number of anatomical body joints extracted from pose estimation. The framework uses a dual ResNet convolutional encoder to learn discriminative representations of the spatial information from both optical flow and skeletal modalities. These encoders produce the spatial feature embedding $F_{s}^{o}$ and $F_{s}^{k}$ that have dimension $d_{s}$, via which motor coordination features, such as joint displacement irregularities, gait instability, posture asymmetry, and limb synchronization inconsistencies, can be extracted. Sequential movement continuity and long-range temporal dependency are modeled by adding Transformer encoders with multi-head self-attention to the architecture. The Transformer module learns temporal embeddings $F_{t}^{o}$ and $F_{t}^{k}$ of the temporal dimension $d_{t}$, effectively capturing inter-frame coordination patterns, the evolution of movement trajectories, and dynamic posture transitions throughout the entire motion sequence. Then, a cross-modal attention fusion mechanism is used to learn an optical flow temporal representation and a skeletal temporal representation, and to fuse them adaptively, generating a multimodal embedding $F_{u}$ that captures both articulated body motion coordination and optical flow information. This adaptive fusion enhances resistance to inter-subject motor variability, motion noise, occlusion, and inconsistent skeletal estimation. Lastly, the unified feature representation is fed into fully connected classification layers, which enable Softmax-based probabilistic prediction of motion activity classes through multimodal spatiotemporal feature representation. The proposed HCT framework enables accurate, interpretable, and clinically reliable assessment of neurodevelopmental motor disorders from motion imaging sequences by integrating spatial learning, temporal dependency modeling, and adaptive multimodal fusion. As discussed, the overall architecture of this HCT Framework is shown in Figure 1.

The HCT framework performs automated detection of DCD with a unified learning architecture that leverages both motion dynamics and skeletal coordination analysis from motion imaging sequences, as illustrated in Figure 1. First, the input video frames are pre-processed, and in this part, dense optical flow maps and skeletal pose sequences are extracted to obtain complementary movement features for representation generation. These modalities undergo further processing via several parallel ResNet-based CNN encoders to learn discriminative spatial features associated with posture stability, limb coordination, and movement irregularities. Then, Transformer-based temporal dependency modules utilize self-attention mechanisms to capture long-range sequence correlations over multiple motion frames for better temporal representation learning. Finally, a cross-modal attention fusion strategy combines flow and skeletal embeddings into a common diagnostic representation, which is then fed to the classification head to recognize DCD-related motor impairments precisely.

### 3.1. Motion Imaging Sequence Acquisition and Pre-Processing

The initial phase of the HCT framework involves capturing and pre-processing motion imaging sequences into a standardized, discriminating input for automated identification of DCD. Motion videos were recorded during motor assessments, including walking, balancing, jumping, transferring objects, maintaining posture, direction changes, hand transitions, and coordination between visual perception and hand movements during object execution. Motion blur, camera instability, illumination variations, and background clutter are common in raw motion recordings, necessitating multiple pre-processing steps to improve feature consistency before deep feature learning and thereby enhance the quality of spatiotemporal representations. The captured motion video is signified as a temporal frame sequence that is denoted as $V=\{F_{1},F_{2},F_{3},\ldots ,F_{T}\}$, where V is denoted as a motion image sequence, $F_{t}$ is represented as the video frame at temporal index, and captured frames are represented as T. Every collected $F_{t}$ has spatial details linked to limb orientation, body posture, motor coordination patterns, and gait. The gathered raw frames differ due to various camera settings and acquisition environments. Therefore, every $F_{t}$ is resized into a fixed resolution (H×W) to perform the spatial normalization, which is represented as $F_{t}^{\prime }\in R^{H\times W\times C}$; here, H is image height, W is image width, and the color channel dimension is represented as C. Afterwards, histogram normalization is applied to reduce illumination inconsistencies and improve contrast uniformity across frames. The intensity distribution of the normalized frame is denoted as $I_{n}(x,y)=\frac{I(x,y)-I_{min}}{I_{max}-I_{min}}$, which is computed from the original pixel intensity (I(x,y)), minimum ($I_{min}$) and the maximum value of the intensity ${(I}_{max})$. The normalization process increases robustness and minimizes lighting variability due to environmental illumination fluctuations during movement acquisition. In addition, gaussian filtering ($G(x,y)=\frac{1}{2\pi \sigma^{2}}e^{-\frac{x^{2}+y^{2}}{2\sigma^{2}}}$) is applied to minimize the occurrence of high-frequency noise and motion artifacts due to rapid body movement and sensor instability. By convolving the image frame with a Gaussian kernel, we obtain a filtered frame that suppresses high-frequency noise while preserving important motion boundaries and posture structures. Since background objects and environmental litter might hinder the learning of movement representation, background subtraction is used to extract foreground movement. The notation of the foreground motion mask is $M_{t}(x,y)=|F_{t}(x,y)-B(x,y)|$; here, B(x,y) is the background frame, and foreground motion intensity is denoted as $M_{t}(x,y)$. Along with this, thresholding is applied to separate the human movement region according to this condition: $S_{t}(x,y)=\left\{\begin{array}{cc} 1, & M_{t}(x,y)>\tau \\ 0, & \mathrm{otherwise} \end{array}\right.$, where τ is the motion threshold value. Then, uniform temporal representation between motion recordings is managed by applying the temporal frame sampling, and the standard sample sequence is represented as $S=\{I_{1},I_{2},I_{3},\ldots ,I_{N}\}$. Uniform temporal sampling preserves similar distributions of frames across all motion sequences, independently of the original video duration, thereby improving learning stability during CNN- and Transformer-based spatiotemporal feature extraction. This pre-processing then produces motion sequences that are denoised, spatially normalized, temporally aligned, and foreground-enhanced for optical flow estimation, skeletal pose extraction, location-to-shape-based spatial feature learning, and long-range temporal dependency modeling in the subsequent stages of the introduced HCT framework. According to the discussions, the pre-processed sample input frames are shown in Table 1.

Table 1 presents the outputs at each pre-processing step of Phase 1 under the proposed HCT framework, using motion frames sampled from the NTU RGB+D 120 dataset. First, raw RGB motion frames encompassing various human movement activities are extracted from unbroken videos. The frames are then resized to a fixed spatial resolution, ensuring that all motion samples share the same dimensions for consistent deep feature learning. The input image undergoes intensity normalization to reduce illumination variation and improve contrast homogeneity, followed by Gaussian filtering that suppresses motion noise and high-frequency artifacts while preserving important posture boundaries. In addition, background subtraction is used to generate binary motion masks and foreground-extracted movement representations, further distinguishing human motion regions from uneventful or irrelevant environmental information. Finally, temporal frame sampling results in a global distribution of body motions across all frames throughout the motion sequence, which facilitates robust optical flow estimation, skeletal pose extraction, and spatiotemporal feature learning (gathering information over both space and time) at later stages of the HCT architecture.

### 3.2. Optical Flow Generation

**Figure 2:**
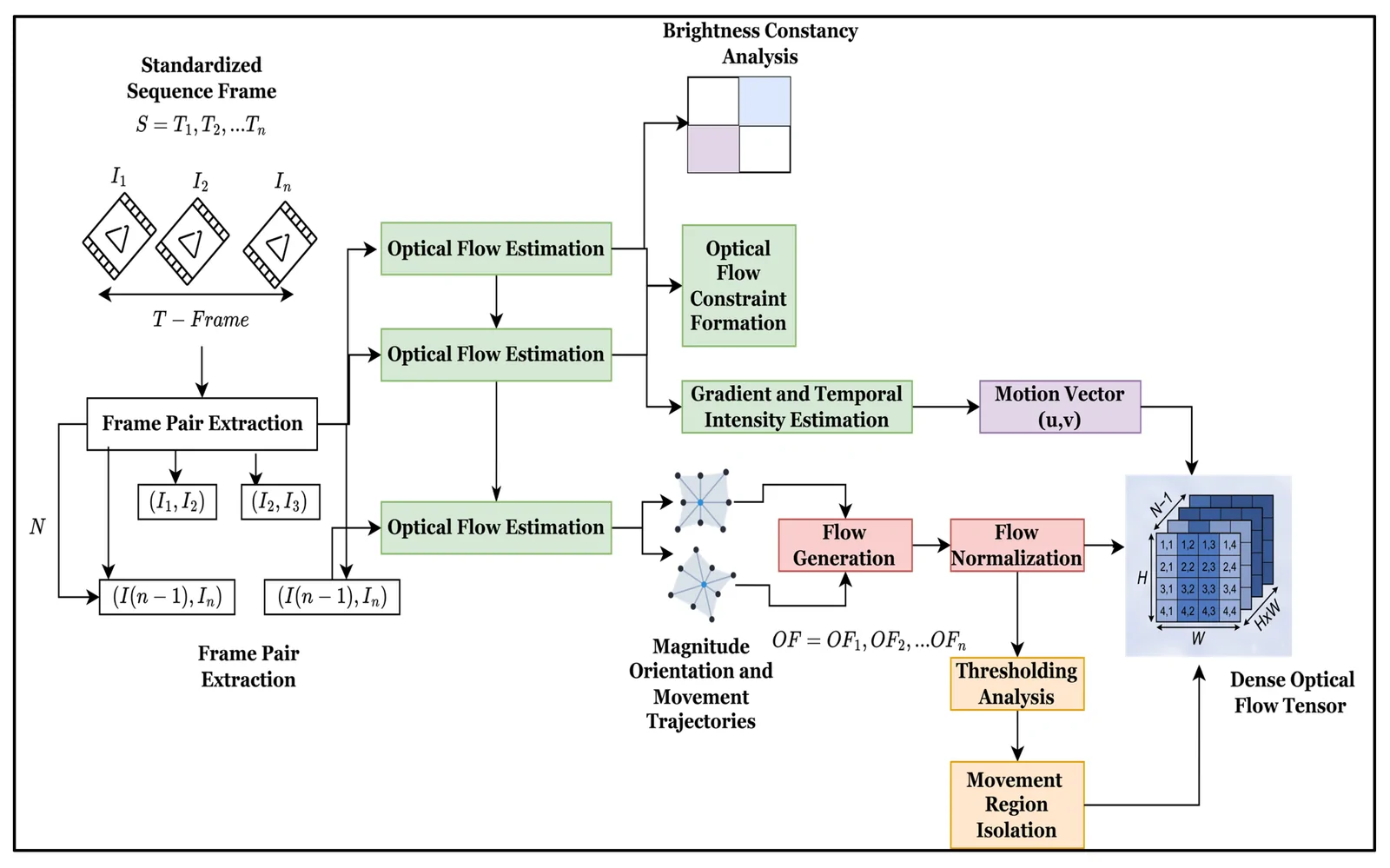
Process of optical flow generation.

The optical flow representations generated by the proposed HCT framework, based on pre-processing and temporal sampling steps, capture dense motion dynamics, including directional information that describes movement behavior across sequential frames during infinite motion imaging. Optical flow is vital to neurodevelopmental motor assessment, as it captures pixel-level displacement between consecutive frames and can reflect movement continuity, gait variability, balance stability or instability, and abnormal limb coordination in children with DCD. In contrast to static frame analysis, optical flow retains temporal motion information by encoding the size and direction of movement paths across adjacent time intervals, thereby improving sensitivity to subtle motor abnormalities that may be relevant in pediatric movement sequences. The structure of the optical flow generation procedure is shown in Figure 2.

Let $I_{t}$ and $I_{t+1}$ be two consecutive frames (as shown in Figure 2) extracted from the sampled motion sequence. In optical flow estimation, we assume the intensity of a moving pixel changes only slightly between adjacent (in time) frames, even though the pixel may be several pixels apart in position. This assumption of brightness constancy can be expressed mathematically with the help of spatial position pixel intensity (I(x,y,t)), horizontal (Δx), and vertical (Δy) pixel displacement and temporal variation between the consecutive frames (Δt), which is defined as I(x,y,t)=I(x+Δx,y+Δy,t+Δt). The first-order Taylor series is applied to compute the motion displacement, which is done by applying it to brightness constancy with the optical flow constraint that is defined as $I_{x}u+I_{y}v+I_{t}=0$. Here, the spatial image gradients in the horizontal and vertical directions are denoted as $I_{x}\;and\;I_{y}$. Temporal intensity variation between frames is represented as $I_{t}$, and motion velocities in the horizontal and vertical components are represented as u and v. So, the motion vector $\left(u,v\right)$ signifies a displacement field defined by pixel trajectories between two frames. The dilation of these displacement vectors generates dense optical flow maps that encode directionality in body motion and stance changes throughout the motion sequence. Within the proposed framework, optical flow estimation is calculated for all inline consecutive frame pairs in the canonical motion sequence S, which is defined as $S=\{I_{1},I_{2},I_{3},\ldots ,I_{N}\}$; here, N is represented as the temporal sequence length. From this, the generated optical flow is defined as $OF=\{OF_{1},OF_{2},OF_{3},\ldots ,OF_{N-1}\}$*,* where $OF\in R^{N\times H\times W\times 2}$. The $OF_{t}$ is generated between the $I_{t}\;\mathrm{and}\;I_{t+1}$ frames that encode various diagnostic motor characteristics, such as limb displacement continuity, sequential gait consistency, body balance instability, posture transition, and inter-limb coordination transition. To perform normalization of flow, since raw optical flow vectors can be affected by illumination changes, camera shake, or smooth changes in body motion, noise can persist; hence, motion representation magnitudes may not be consistent across all sequences. Then the normalized motion magnitude is defined as $M(x,y)=\sqrt{u(x,y{)}^{2}+v(x,y{)}^{2}}$, and the insignificant background motion and noise artifacts are removed by applying the motion thresholding that is defined as $T(x,y)=\left\{\begin{array}{cc} 1, & M(x,y)>\tau \\ 0, & M(x,y)\leq \tau \end{array}\right.$; here, τ is denoted as the threshold parameter that helps to separate the significant movement region. The optical flow maps are then converted into dense motion tensors, which are passed to the ResNet-based convolutional encoder for discriminative spatial feature extraction. By generating optical flow representations of the raw data that preserve both directional movement trajectories and temporal displacement continuity, the proposed motion capture (HCT) framework can robustly discern scabietic features in fine motor coordination associated with DCD and improve automated neurodevelopmental diagnostic performance. According to the discussion, the generated optical flow representation is shown in Table 2.

The generated frames show the conversion of temporal motion states to dense, activity-specific representations of body movement features over time. There are spatial velocity variations, such as these, that physically induce the range of motion required for body posture transitions and limb movement execution in directional displacement information, as shown using color-encoded optical flow maps. In regions with greater motion activity, we can more confidently determine temporal transitions to increased frequency, enabling precise identification of coordinated and uncoordinated movement behavior within individual segment/frame intervals across the overall motion sequence. Simultaneously, the motion magnitude representations focus on velocity-intensity distributions by highlighting distinct areas of activity related to posture changes, gait progression, and changes in trajectory across limbs. These representations improve the sensitivity to motion in the temporal domain and diminish reliance on static appearance information, suggesting the potential of the proposed HCT framework to extract detailed spatiotemporal features sufficient to detect subtle coordination synergies and sequential motor irregularities for DCD assessment.

### 3.3. Skeletal Pose Generation

**Figure 3:**
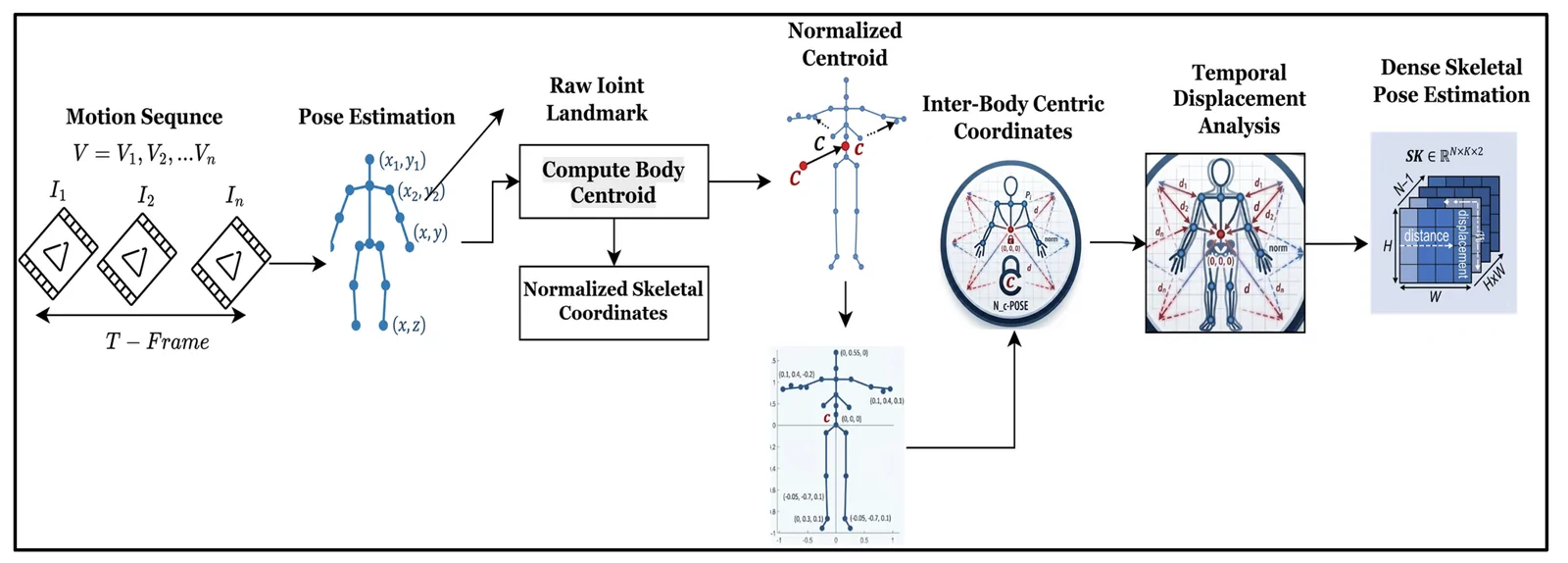
Flow of skeletal pose generation.

After optical flow computation, the proposed HCT framework generates a skeletal pose representation to extract articulated body coordination structures and the joint movement dependencies from motion image sequences. Optical flow captures dense motion information but does not explicitly model anatomical body configuration and inter-joint coordination, which are required to capture subtle irregularities in the motor behavior of DCD. In this regard, skeletal pose estimation is integrated to obtain biomechanically interpretable representations of movement that capture posture alignment, balance consistency, limb synchrony, and trajectory continuity during motor activity. The skeletal pose generation process is shown in Figure 3.

Each pre-processed motion frame ($I_{t}$) is first fed into a pose estimation network to estimate anatomical joint locations using large body landmarks (shoulders, elbows, wrists, hips, knees, ankles). The extracted skeletal representation ($P_{t}$) for every frame is represented as $P_{t}=\{(x_{1},y_{1}),(x_{2},y_{2}),\ldots ,(x_{K},y_{K})\}$, where K is the detected body points and $\left(x_{k},y_{k}\right)$ represents the spatial coordinates at joints. Hence, the entire skeletal sequence of motion video is signified as $SK=\{P_{1},P_{2},P_{3},\ldots ,P_{N}\}$, where N is the temporal frame sequence length. Body joint coordinates are normalized with respect to the body centroid to make the system more robust against scale and camera distance variations, as well as different subject positions. The centroid coordinates are calculated using Equation (1).

(1) xc=1K∑k=1Kxk,yc=1K∑k=1Kykx^k=xk−xcW,y^k=yk−ycH

**Figure 4:**
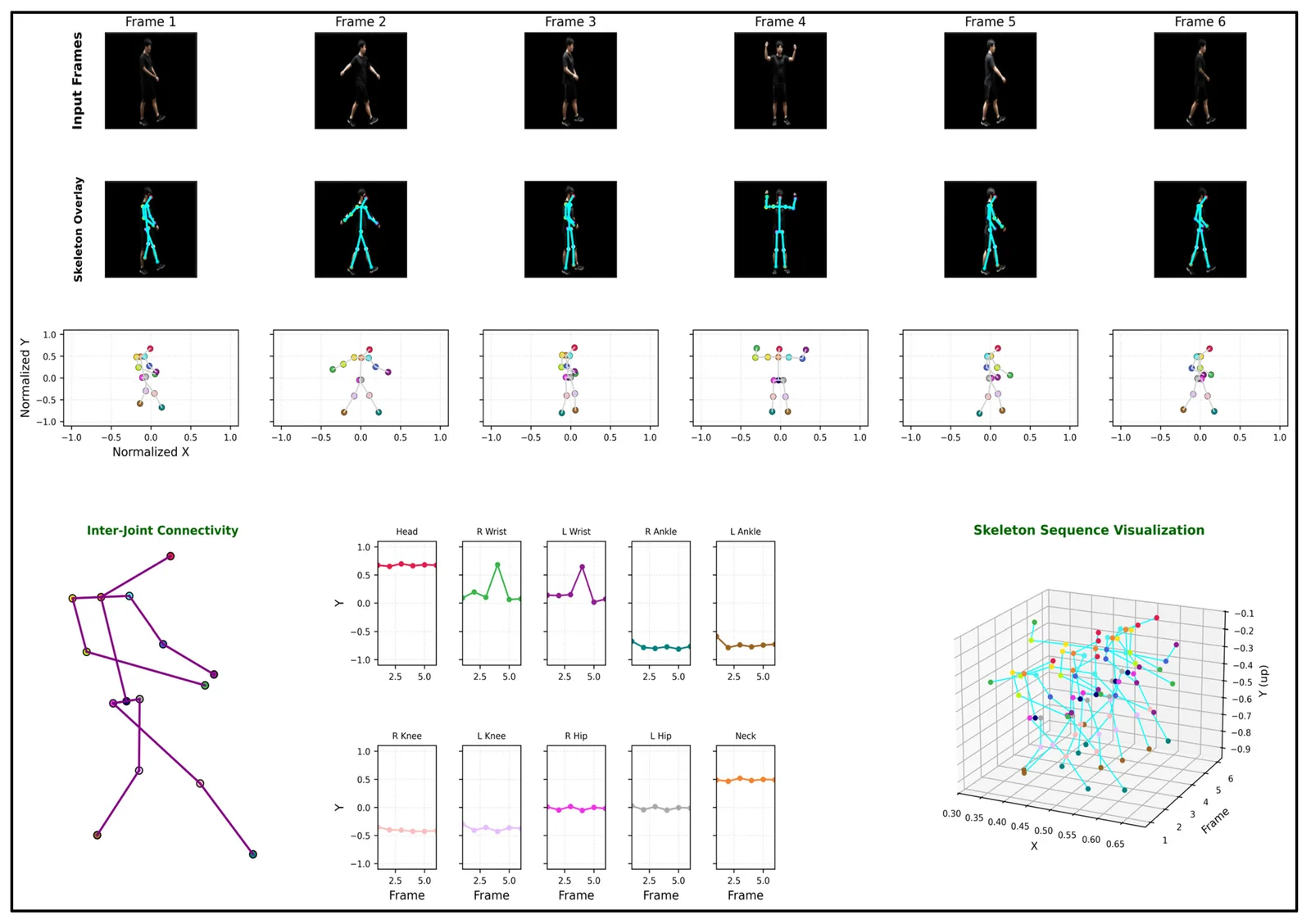
Sample outputs for skeletal pose embeddings.

According to Equation (1), the normalization process confirms that the skeletal illustration remains invariant to camera positioning, environmental acquisition conditions, and body scale. Then the created skeletal tensor is signified as $SK\in R^{N\times K\times 2}$; the temporal frame count is denoted as N, and the anatomical joint count is denoted as K. In addition, the inter-joint Euclidean distance relationship between the body points is estimated to characterize the body coordination dynamics, as described by $D_{ij}=\sqrt{(x_{i}-x_{j}{)}^{2}+(y_{i}-y_{j}{)}^{2}}$. The computed $D_{ij}$ gives the discriminative information about limb extension, posture symmetry and joint coordination irregularities. The model movement continuity is estimated by computing the temporal joint displacement, which is estimated as $\Delta P_{k}^{t}=\sqrt{(x_{k}^{t+1}-x_{k}^{t}{)}^{2}+(y_{k}^{t+1}-y_{k}^{t}{)}^{2}}$; here, displacement variation is denoted as $\Delta P_{k}^{t}$ of the $k^{th}$ joint. The skeletal pose representations captured in this phase will capture articulated motion structure, sequential body coordination dependencies, and biomechanical posture transitions associated with motor execution behavior. The derived skeletal pose embeddings outputs are shown in Figure 4.

These interpretable joint-level representations (as shown in Figure 4) are used to complement dense optical flow dynamics and then fed to the ResNet-based spatial feature extraction module to perform discriminative spatiotemporal learning in the proposed HCT framework.

### 3.4. ResNet-Based Spatial Feature Extraction

**Figure 5:**
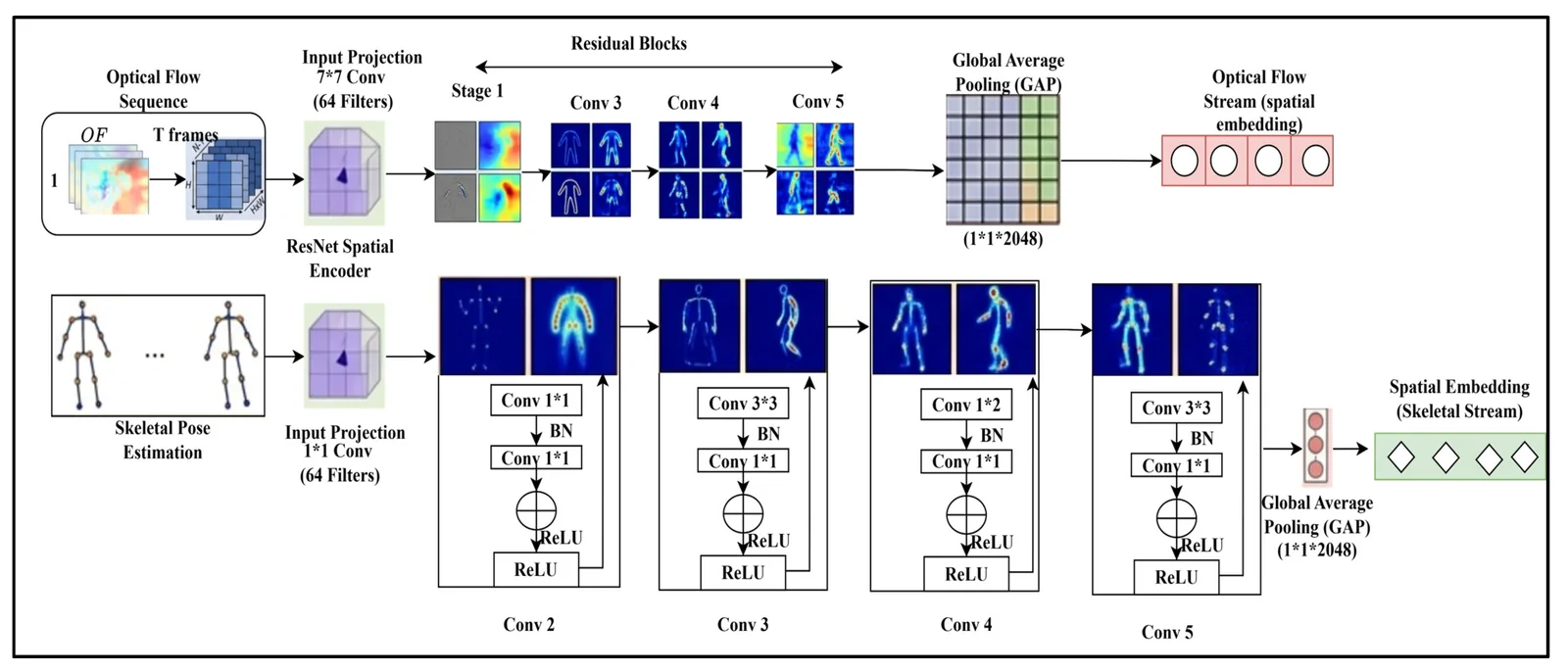
Layer-wise illustration of ResNet-based feature extraction.

After generating the skeletal pose models, the proposed HCT framework extracts the spatial features discriminatively through a ResNet-based convolutional architecture. The main aim of this stage is to train the model to represent the hierarchical movement patterns from optical flow maps and skeletal motion structures to detect posture instability, joint coordination irregularity, gait asymmetry, and inconsistency of movement trajectory commonly found in individuals with DCD. The proposed framework utilizes the residual learning mechanisms to maintain the gradient propagation and enhance the spatial representation learning capacity over complex motion patterns, while preserving feature information during the optimization of deeper networks, contrary to the conventional shallow CNN architecture that would lead to feature degradation. The feature extraction structure is shown in Figure 5.

The optical flow tensor OF and the skeletal representation tensor SK are initially represented in $R^{N\times H\times W\times 2}$ and $R^{N\times K\times 2}$, respectively. Convolutional transformations are applied to each input sequence, with learnable kernels designed to extract local spatial descriptors relevant to motion orientation, limb positioning, body posture, and/or inter-joint structural dependency patterns. The convolutional transformation $Z_{l}\;$ is applied to an input feature map X. The convolution process uses the kernel weights ($W_{l}$), bias parameters ($b_{l}$), non-linear activation function (σ), and generated feature map ($Z_{l}$) to process the feature map. The convolutional layers progressively learned low- to high-level motion features, including posture contours, edge structures, coordination, and gait representations. However, gradient vanishing and optimization instability often result from increasing the network’s depth. To overcome this drawback, residual skip connections are added to the ResNet encoder. Residual learning mechanism is represented as H(x)=F(x)+x. This residual mapping allows motion information to be passed directly between layers, thereby retaining discriminative information and improving network convergence. The ResNet encoder is a composite module composed of a series of residual blocks, each consisting of multiple convolutional layers, batch normalization, a Rectified Linear Unit (ReLU), and an identity shortcut connection. The batch normalization is done for the purpose of stabilizing the distribution of features across iterations of training and is computed using Equation (2).

(2) $$BN(x)=\gamma \left(\frac{x-\mu_{B}}{\sqrt{\sigma_{B}^{2}+\epsilon }}\right)+\beta$$

In Equation (2), the batch mean and variance are denoted as $\mu_{B}$ and $\sigma_{B}^{2}$, learnable scaling and shifting parameters are represented as γ and β, and the small stabilization constant is denoted as ϵ. In addition to this, the ReLU activation function is utilized to minimize the negative feature activations in the process, and is defined as ReLU(x)=max(0,x). This phase extracts spatial feature maps that include limb coordination irregularities, posture imbalance patterns, joint displacement inconsistencies, body alignment instability, trajectory deviations, and gait asymmetry. To minimize the spatial dimensionality while preserving movement information, global average pooling is applied to the feature map defined in Equation (3).

(3) $$\left.\begin{array}{c} GAP\left(Z\right)=\frac{1}{H\times W}\sum_{i=1}^{H}\sum_{j=1}^{W}Z(i,j)\\ F_{s}^{o}\in R^{N\times d_{s}},F_{s}^{k}\in R^{N\times d_{s}} \end{array}\right\}$$

In Equation (3), $F_{s}^{o}$ is represented as the flow spatial features, skeletal spatial features are represented as $F_{s}^{k}$, and spatial embedding dimensionality is denoted as $d_{s}$. These discriminative embeddings are spatially coordinated and capture local irregularities in motion, which are essential for DCD-based motion analysis. The extracted spatial features are then passed to the Transformer-based temporal dependency learning module to model long-range sequential dependencies in the proposed HCT framework. According to the discussions, the statistical derivation for spatial features is shown in Table 3.

The statistical feature outputs (Table 3) indicate that the ResNet-based spatial feature extraction module gradually learns discriminative representations of motion coordination steps across both optical flow and skeletal pose modalities. Frames with higher mean flow activation and maximum response values reflect the intensity and size of limb movement areas, and they suggest that the convolutional encoder effectively highlights dynamically active posture changes and gait variations in the motion sequence. At the same time, high values of skeleton activation and joint coordination variance indicate greater articulated body dependency learning, especially during the complex stages of movement that involve imbalance correction, upper-body transition, and alterations in inter-limb synchronization. The gradual change in spatial feature entropy across frames also suggests adaptive hierarchical representation learning, with deeper residual layers learning increasingly complex spatial dependencies and motion irregularity structures beyond those captured by edge or contour features alone. Such learned embeddings thus exhibit strong spatial discrimination, as they retain not only dense motion-sensitive parts of the body but also articulated skeletal coordination patterns, which enable the reliable extraction of diagnostically relevant motor features for subsequent analysis by a Transformer-based temporal dependency in the proposed HCT framework.

### 3.5. Transformer-Based Temporal Dependency Learning

**Figure 6:**
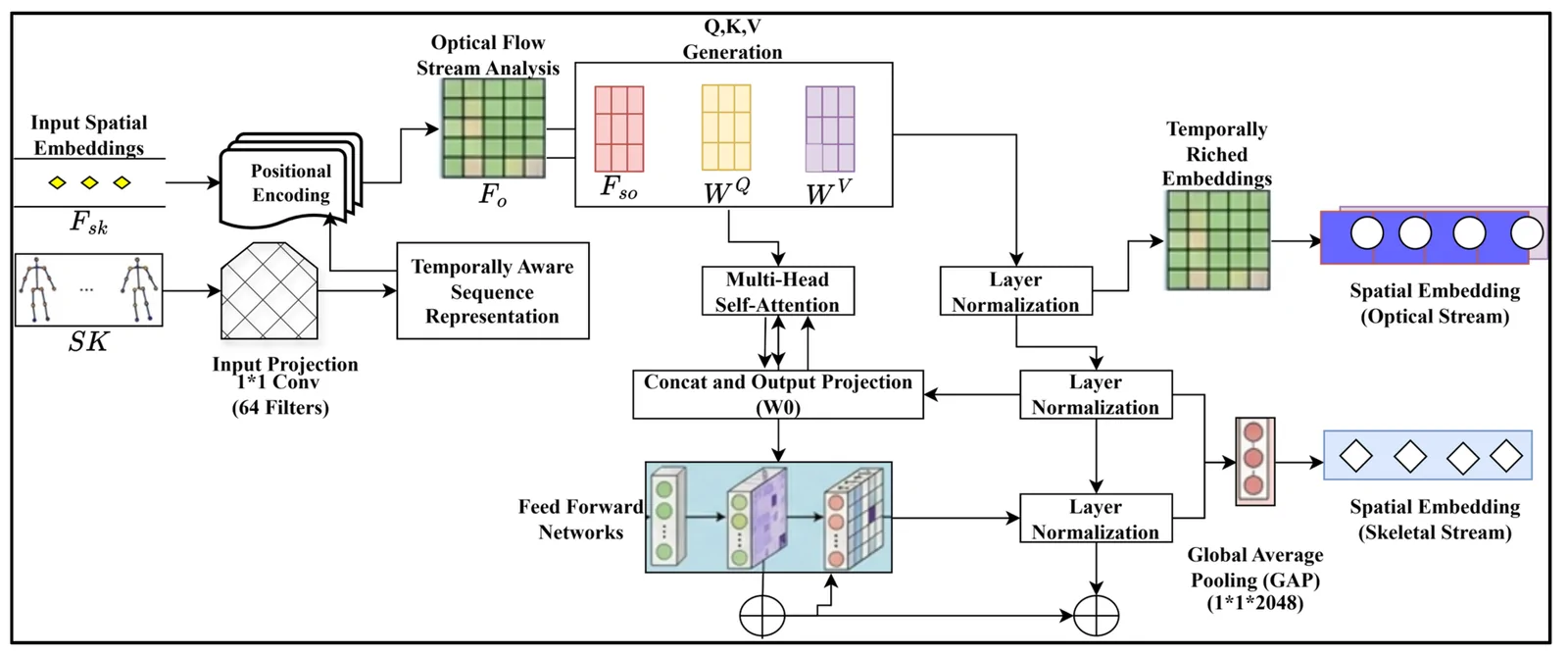
Process of temporal dependency learning.

Following the extraction of discriminative spatial embeddings with the ResNet encoder, the proposed HCT framework uses Transformer-based temporal dependency learning to learn long-range sequential coordination relationships between motion imaging sequences. The phase aims to capture temporal continuity, movement evolution, posture transitions between frames, and sequential motor dependency patterns related to DCD. Even though convolutional spatial learning is effective in maintaining local movement properties, it is still inadequate to describe the lengthy time coordination behavior that is spread over several motion frames. Thus, a multi-head self-attention Transformer encoder is introduced to capture global contextual dependencies between temporally distinct movement representations. The structure of temporal dependency learning is shown in Figure 6.

Consider the optical flow and skeletal streams, denoted as $F_{s}^{o}=\{f_{1}^{o},f_{2}^{o},\ldots ,f_{N}^{o}\}$ and $F_{s}^{k}=\{f_{1}^{k},f_{2}^{k},\ldots ,f_{N}^{k}\}$, which are processed with the help of a transformer architecture. The transformer uses positional encoding to maintain temporal frame information across the motion sequence. Then the positional encoding operation is signified as Equation (4).

(4) $$\left.\begin{array}{c} PE\left(pos,2i\right)=\sin\left(\frac{pos}{10000^{2i/d}}\right)\\ PE(pos,2i+1)=cos\left(\frac{pos}{10000^{2i/d}}\right) \end{array}\right\}$$

In Equation (4), the temporal frame position is denoted as pos with dimension index (i) and embedding dimensionality (d). The derived positional information is combined with spatial embedding to create a temporally aware sequence suitable for self-attention learning. For every input embedding, the query, key, and value matrices are generated as $Q=FW_{Q},K=FW_{K},V=FW_{V}$, and the respective global temporal relationship is estimated with the help of the scaled dot product that is estimated as $Attention(Q,K,V)=Softmax\left(\frac{QK^{T}}{\sqrt{d_{k}}}\right)V$. The attention mechanism assigns weights to temporal positions to identify irregularities in sequential coordination and movement transitions within the motion sequence. Then the multi-head attention is utilized to improve diversity representation, which is defined in Equation (5).

(5) $$\left.\begin{array}{c} MH\left(Q,K,V\right)=Concat\left(head_{1},\ldots ,head_{h}\right)W_{O}\\ head_{i}=Attention(Q_{i},K_{i},V_{i}) \end{array}\right\}$$

**Figure 7:**
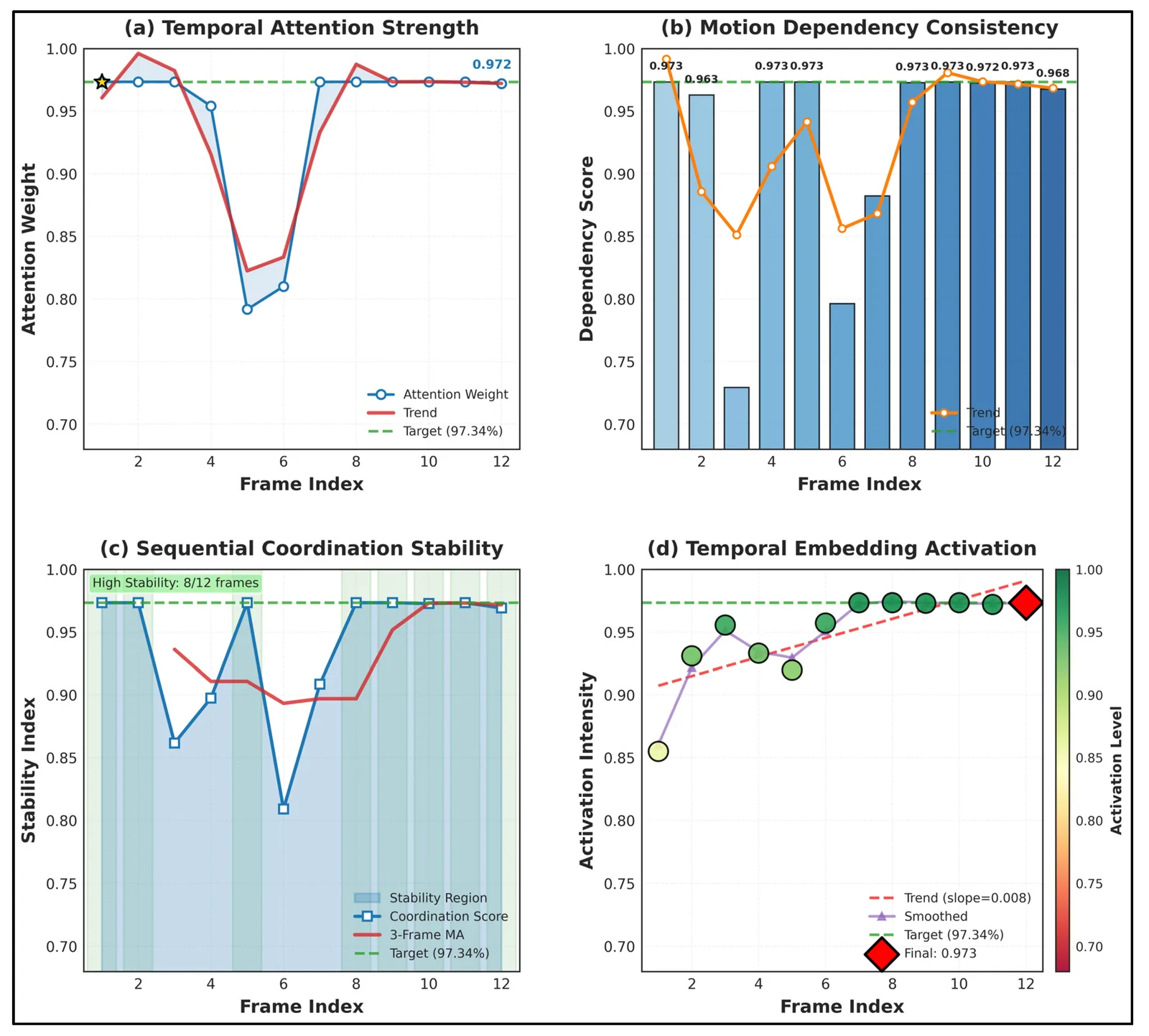
Graphical analysis of transformer-based temporal dependency learning analysis.

The computed multi-head attention enables learning temporal dependencies related to posture evolution, gait continuity, and sequential movement consistency. Along with this, feed-forward transformation is applied to enhance temporal feature representation that is defined as $FFN(x)=\sigma (xW_{1}+b_{1})W_{2}+b_{2}$. Furthermore, layer normalization and residual skip connection are utilized to stabilize the optimization and maintain the feature continuity in the transformer layer; this is computed as $LN(x)=\frac{x-\mu }{\sqrt{\sigma^{2}+\epsilon }}$. According to this process, the temporally enhanced embeddings are defined as $F_{t}^{o}\in R^{N\times d_{t}}\;and\;F_{t}^{k}\in R^{N\times d_{t}}$. These time-based embeddings have long-range sequential coordination features such as gait continuity, posture change dynamics, movement consistency, and inter-frame motor dependency structures. The resulting representations consequently fully characterize the temporal movement behavior of pediatric patients and are then fed to the cross-modal attention fusion unit to generate unified multimodal diagnostic embeddings in the proposed HCT system. According to the analysis, the impact of this phase is illustrated in Figure 7.

Figure 7 shows the results from the temporal dependency learning stage of the proposed HCT framework, trained with the temporal learning module, achieving an overall accuracy of 97.34%. Figure 7a visualizes the temporal attention strength $A_{ij}$ between source and target frames, and it is shown that the self-attention mechanism can learn the long-range dependency in sequential motion frames. Progress towards the target accuracy, as reflected in increasing accuracy over time, suggests greater contextual understanding of gait progression and posture transition patterns. The motion dependency consistency score $D_{c}$ in Figure 7b represents the consistency of the inter-frame temporal relationships among the images. The dependency value in later frames is higher, further demonstrating that the Transformer encoder can maintain movement continuity and learn coordinated information from optical flow and skeleton embeddings that convey meaning over time. The index of sequential coordination stability ($C_{s}$) is shown in Figure 7c and measures the consistency of feature propagation over time in the motion sequence. The moving-average curve shows reduced fluctuations during dynamic movement transitions, balance adaptation, and limb synchronization, indicating stable coordination learning. Figure 7d depicts the temporal embedding activation $E_{a}$, which is the strength of the sequence-aware contextual embeddings produced by multi-head self-attention. The progressive activation intensity shows the evolution of temporal representations of features, enabling the proposed HCT framework to model complex movement evolution and motor coordination behavior necessary for the accurate identification of DCD.

### 3.6. Cross-Modal Attention Fusion and Prediction

**Figure 8:**
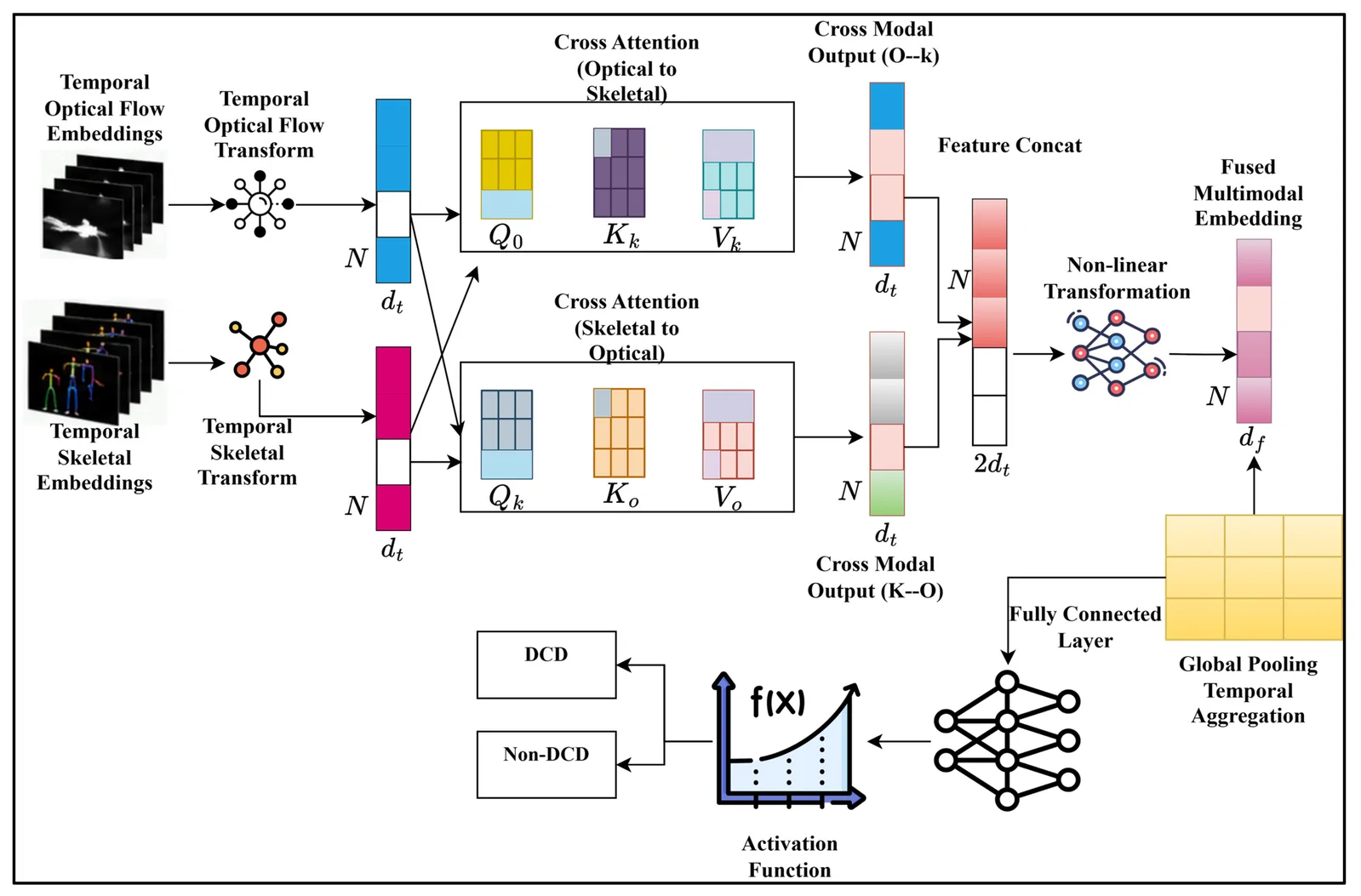
Process of fusion and DCD prediction.

The proposed HCT framework first extracts temporally enriched embeddings with a Transformer encoder and then fuses complementary motion information from optical flow and skeletal pose using a cross-modal attention mechanism. The optical flow stream can accurately describe dense motion displacement and movement direction, but is not explicit about articulated body structure information. In contrast, skeletal embeddings capture relationships between joints and posture configurations, but may lack details of motion continuity. Hence, cross-modal fusion is proposed to integrate both representations, enabling a unified discriminative embedding that models the full spatiotemporal motor behavior of DCD. The process is shown in Figure 8.

The optical flow and skeletal branches are used to create the temporal embeddings as $F_{t}^{o}\in R^{N\times d_{t}}\;and\;F_{t}^{k}\in R^{N\times d_{t}}$, where temporal flow embedding is denoted as $F_{t}^{o}$ and skeletal embedding is $F_{t}^{k}$. For learning inter-modal dependency relationships, a cross-attention mechanism is used between the two feature streams. Optical flow embedding is used as the query representation, whereas a skeletal embedding is used as the key and value representations (defined in Equation (6)).

(6) $$\left.\begin{array}{c} Q_{o}=F_{t}^{o}W_{Q}\\ K_{k}=F_{t}^{k}W_{K}\\ V_{k}=F_{t}^{k}W_{V} \end{array}\right\}$$

From this, the crossmodal attention is estimated as Equation (7).

(7) $$CA(Q_{o},K_{k},V_{k})=Softmax\left(\frac{Q_{o}K_{k}^{T}}{\sqrt{d_{k}}}\right)V_{k}$$

In Equation (7), the key embedding dimensionality is denoted as $d_{k}$. The attention process ensures that the framework effectively predicts movement pattern correlations between articulated skeletal coordination and dense motion trajectory structures. Similarly, reverse cross attention is computed with the help of the skeletal embeddings (query) and optical flow embeddings (key–value pair), which are represented as $CA(Q_{k},K_{o},V_{o})$. Further, bidirectional interactions enhance complementary features, improve robustness, and address posture inconsistency. Finally, the fused multimodal embeddings are generated via a non-linear transformation and feature concatenation, which is defined as $F_{c}=\sigma (F_{f}W_{f}+b_{f})$. The framed $F_{c}\in R^{N\times d_{f}}$ ensures joint coordination dependency, dense motion continuity, posture evolution, temporal movement, and gait trajectory consistency. The cross-modal attention fusion stage uses adaptive attention to integrate optical flow and temporal motion representations from the skeleton, producing powerful motor embeddings that capture subtle coordination abnormalities and irregularities characteristic of DCD. The integrated representations are then passed to the final classification block for automated prediction and diagnosis of neurodevelopmental disorders (NDDs) in the proposed HCT framework.

**Figure 9:**
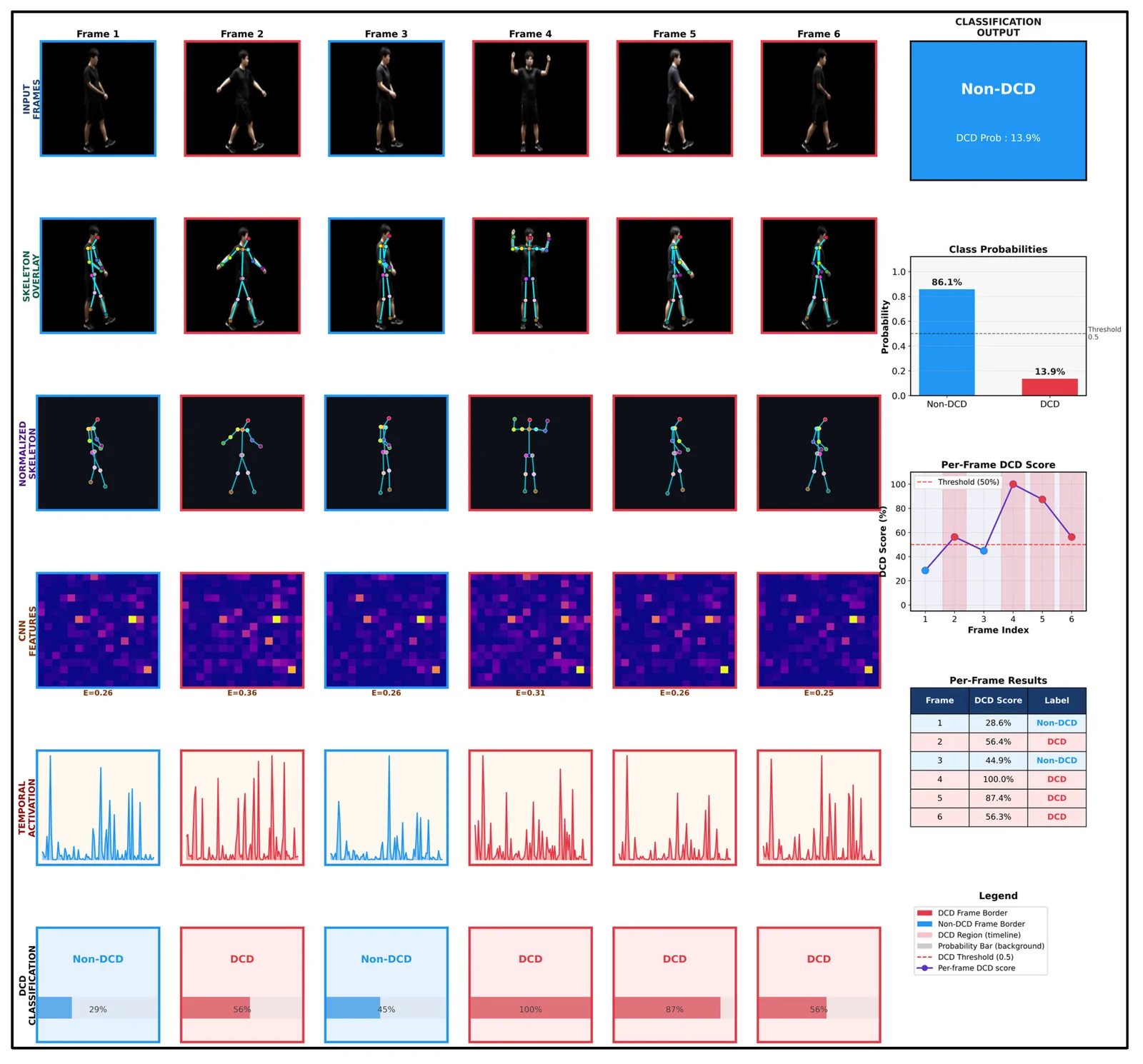
Final output representation of the HCT framework.

The proposed HCT framework, after creating a unified multimodal embedding via cross-modal attention fusion, performs automatic classification and diagnostic prediction for DCD detection. In this stage, the fused temporal representation $F_{c}\in R^{N\times d_{f}}$. The integrated motion continuity, skeletal coordination, posture evolution, and long-range temporal dependency information are passed to a fully connected classification network for discriminative learning. The classifier gradually maps the multimodal embedding to a low-dimensional representation of diagnostic features that captures the coordination irregularities seen in DCD. Dense layers and activation functions are used to highlight movement characteristics relevant to the diagnosis, suppress responses to redundant features, improve separability between non-linear features, and increase classification robustness. Then, a Softmax prediction layer generates normalized probability distributions for DCD and non-DCD categories, enabling confidence-aware diagnostic decisions. The cross-entropy loss minimization technique is used during model optimization to reduce the prediction error between clinical labels and the classification probabilities generated by the model, thereby generalizing across various patterns of pediatric movement. The detailed process is shown in Algorithm 1. According to the discussions, the final outputs are shown in Figure 9.

**Algorithm 1:** Hybrid CNN–Transformer (HCT) Framework for Multimodal Spatiotemporal Representation Learning
**Input:** $F=\{f_{1},f_{2},\ldots ,f_{N}\}$: Sequence of RGB video frames**Output:** $C_{pred}$: Predicted movement class
**Begin**

    1. **for** i=1 **to** N **do**    2.      $f_{i}\leftarrow \mathrm{Resize}(f_{i},224\times 224)$    3.      $f_{i}\leftarrow \mathrm{Normalize}(f_{i})$    4. **end for**    5. **for** i=1 **to** N−1 **do**    6.      $O_{i}\leftarrow \mathrm{RAFT}(f_{i},f_{i+1})$ // Optical flow estimation    7. **end for**    8. **for** i=1 **to** N **do**    9.      $P_{i}\leftarrow \mathrm{MediaPipe}(f_{i})$ // Pose estimation    10.    $P_{i}\leftarrow \mathrm{ZScoreNormalize}(P_{i})$    11.  **end for**    12.  **for** i=1 **to** N−1 **do**    13.    $S_{i}\leftarrow \mathrm{ResNet}(O_{i})$ // Spatial feature extraction    14.  **end for**    15.  $T\leftarrow \{S_{1},S_{2},\ldots ,S_{N-1}\}$    16.  $T\leftarrow \mathrm{LinearProjection}(T)+E_{pos}$    17.  **for** l=1 **to** L **do**    18.    T←MultiHeadSelfAttention(T)    19.    T←FeedForward(T)    20.    T←LayerNormalization(T)    21.  **end for**    22.  $T_{emb}\leftarrow \mathrm{TemporalEmbedding}(T)$    23.  $F_{fuse}\leftarrow \mathrm{CrossModalAttention}(T_{emb},P)$    24.  $C_{pred}\leftarrow \mathrm{Softmax}(\mathrm{Classifier}(F_{fuse}))$    25.  **Return** $C_{pred}$

**End**



Therefore, the final prediction output provides reliable automated support for neurodevelopmental diagnosis, identifying subtle motor coordination abnormalities, gait inconsistency, posture instability, and movement synchronization deficits, captured throughout the spatiotemporal motion sequence within the proposed HCT framework.

The proposed Hybrid CNN–Transformer (HCT) framework is developed to investigate intelligent multimodal spatiotemporal representation learning for automated movement analysis motivated by motor coordination characteristics associated with Developmental Coordination Disorder (DCD). The NTU RGB+D and NTU RGB+D 120 datasets were selected because they provide synchronized RGB video sequences, optical flow information, and three-dimensional skeletal joint representations captured from diverse human movement activities, enabling comprehensive evaluation of the proposed multimodal learning architecture. The benchmark activities involving gait, posture transitions, balance-related movements, upper- and lower-limb coordination, and whole-body actions provide representative motion patterns for evaluating the robustness of the proposed framework in modeling complex motor behaviors. The reported classification performance demonstrates the computational capability of the proposed architecture in discriminating multimodal movement patterns under standardized benchmark protocols. The study is intended as a computational framework for intelligent motion analysis, while comprehensive clinical validation using expert-confirmed DCD cohorts and standardized neurodevelopmental assessment protocols remains an important direction for future investigation to further establish clinical applicability and generalizability.

## 4. Experimental Setup and Analysis

### 4.1. Implementation Settings

In this section, the implementation environment, data setup, pre-processing, model training configurations, evaluation metrics, and optimization parameters used for validating the presented HCT approach for automatic identification of DCD from motion imaging sequences are described. The experimental design aims to test the efficacy of multimodal spatiotemporal learning across various movement scenarios, while maintaining reproducibility, computational stability, and strong diagnostic generalization. The proposed HCT framework was applied in an environment for large-scale spatiotemporal motion analysis implemented on a GPU-accelerated deep learning framework for multimodal Transformer optimization. The above framework was built using Python 3.10 and PyTorch 2.1 for efficient tensor-based neural network computation and automatic differentiation. The high-throughput training of the ResNet–Transformer architecture and cross-modal attention fusion modules was enabled by GPU parallelization with NVIDIA CUDA 12.1 and an NVIDIA RTX 4090 graphics processor with 24 GB of dedicated memory. The experimental platform also employed an Intel Xeon 3.2 GHz CPU with 128 GB of RAM to ensure stable generation of multimodal embeddings in large-batch mode and processing of high-dimensional features.

The computation of optical flow and the image pre-processing operations were implemented using the OpenCV 4.8 library, which allows the extraction of a dense motion field from successive RGB images, generating a representation of frame-level motion. Using the MediaPipe pose estimation framework, the coordinates of the body landmarks were normalized, representing key joint positions in the human body, such as shoulders, elbows, wrists, hips, knees, and ankles, and the coordinates of the joints were extracted. The numerical computation, matrix transformation, and statistical analysis tasks were implemented using NumPy and SciPy libraries, and visualization and experimental graph production were done with Matplotlib (v3.7.1) and Seaborn (v0.12.2) libraries. The complete framework was implemented on Ubuntu 22.04 LTS, with mixed-precision GPU computation to enhance memory efficiency, reduce Transformer attention-computation latency, and accelerate the optimization of multimodal features in large-scale temporal sequence training.

### 4.2. Dataset Description

To experimentally test the proposed HCT framework, two benchmark motion datasets—NTU RGB+D and NTU RGB+D 120 [34] (https://rose1.ntu.edu.sg/dataset/actionRecognition/, accessed on 26 March 2026)—were used to represent large-scale human action sequences for spatiotemporal movement analysis and articulated motor coordination modeling. The data sets were chosen because they represent a variety of body movement patterns and recording conditions, and offer a range of modalities (RGB, depth, and skeletal pose) that are synchronized for multimodal learning. The NTU RGB+D dataset consists of 56,880 video samples across 60 action classes, collected from 40 subjects, while NTU RGB+D 120 consists of 114,480 video samples across 120 action classes, collected from 106 people. This data includes skeletal models with 25 body joints created from Kinect-based motion capture systems, allowing for the precise modeling of inter-joint coordination behavior, posture transition dynamics, gait continuity, and movement dependencies among body joints. Three synchronized camera viewpoints were recorded at 1920 × 1080 spatial resolution and 30 FPS in indoor environments, enabling robust spatiotemporal movement analysis in multi-view scenarios. The datasets contain a range of motor activities, including walking, sitting, standing, bending, hand movements, transitioning to/from balance, interacting with objects, and coordinated limb execution patterns, all of which are important to consider when examining motor irregularities in DCD. In experiments, RGB frames were used to generate a motion representation of optical flow, and skeletal joint coordinates were used to learn the dependency of the articulated pose. This is due to the dense motion trajectories and the structured skeletal representations, which enabled the proposed HCT framework to perform thorough spatiotemporal feature extraction and long-range temporal coordination modeling in complex human motion sequences.

### 4.3. Hyperparameter Settings

The proposed Hybrid CNN–Transformer (HCT) framework was trained using an optimized set of hyperparameters to ensure stable convergence and effective multimodal spatiotemporal representation learning. The ResNet encoder, comprising residual bottleneck blocks with Batch Normalization and ReLU activation, was employed for spatial feature extraction, whereas the Transformer encoder consisted of 4 stacked encoder layers with 8 multi-head self-attention heads for modeling long-range temporal dependencies. Each input frame was resized to a spatial resolution of 224 × 224 pixels, and the network was optimized using the AdamW optimizer with an initial learning rate of 1 × 10^−4^ and a weight decay of 1 × 10^−5^. A mini-batch size of 16 was selected to balance computational efficiency and gradient stability. The temporal embedding dimension was fixed at 256, while the cross-modal fusion embedding dimension was set to 512 to effectively integrate optical flow and pose-estimation representations. A dropout rate of 0.3 was applied to the Transformer and fusion layers to improve model generalization, and the network parameters were optimized using the cross-entropy loss function. Training was performed for 150 epochs using a cosine annealing learning rate scheduler, together with an early stopping strategy employing a patience value of 15 epochs to prevent overfitting and avoid unnecessary optimization iterations.

The NTU RGB+D and NTU RGB+D 120 datasets were evaluated using the official cross-subject and cross-setup protocols, where the training, validation, and testing subsets were generated using an 80:10:10 partition of the training data while maintaining class-balanced sampling. All experiments were conducted using a fixed random seed (42) to ensure deterministic data partitioning, weight initialization, and mini-batch generation. The proposed Hybrid CNN–Transformer (HCT) framework was independently trained five times, and the reported performance metrics correspond to the mean ± standard deviation together with 95% confidence intervals, providing a statistical assessment of model stability. Hyperparameter selection was performed through validation-based tuning by evaluating multiple candidate learning rates (1 × 10^−3^, 5 × 10^−4^, 1 × 10^−4^), batch sizes (8, 16, 32), dropout rates (0.2, 0.3, 0.5), embedding dimensions (128, 256, 512), and weight decay coefficients (1 × 10^−4^, 1 × 10^−5^).

## 5. Results and Discussions

The comparative performance evaluation of the proposed HCT framework with three recent spatiotemporal movement analysis approaches from the related work, including the MPL-CNN proposed by Shi et al. (2024) [16], the attention-based lightweight CNN proposed by Zhou et al. (2024) [18], and the Spatial–Temporal Transformer model developed by Liang et al. (2025) [21]. The evaluation was performed under various movement conditions, including gait continuity, posture adaptation, balance transition, coordinated walking, arm movement, and sequential motor execution patterns derived from movement sequences captured by motion imaging. All methods were assessed using the same performance metrics (accuracy, precision, recall, F1-score, and specificity) to provide a comprehensive diagnostic assessment. Together, these metrics quantify each framework’s success in maintaining spatiotemporal movement representations that are discriminative and accurately identify motor coordination abnormalities that distinguish DCD. The comparative analysis also shows the impact of multimodal feature integration and long-range temporal dependency modeling on the performance of automated neurodevelopmental diagnosis. The HCT’s efficiency is shown in Table 4.

The proposed Hybrid CNN–Transformer (HCT) framework was evaluated using the NTU RGB+D 120 benchmark dataset, which contains 114,480 RGB video sequences acquired from 106 participants performing 120 human action categories under 32 camera viewpoints. The experiments followed the standard cross-subject and cross-setup evaluation protocols to assess robustness across different individuals and acquisition environments. Each video sequence was converted into synchronized optical flow maps and 25-joint pose-estimation skeletons, enabling multimodal spatiotemporal representation learning. The proposed framework achieved an average classification accuracy of 98.21% under the cross-subject protocol and 97.84% under the cross-setup protocol, demonstrating stable performance under subject and environmental variations. The objective of this study is the development and computational validation of a multimodal spatiotemporal learning architecture rather than direct clinical validation.

The proposed HCT framework can effectively integrate multiple spatial and temporal representation learning, thereby achieving superior performance (Table 4). Let $F_{s}^{o}$ and $F_{s}^{k}$ denote the spatial embeddings learned from the optical flow stream and the skeleton pose stream, respectively, such that the ResNet encoder retains hierarchical motion information via the residual mapping $H\left(x\right)=F\left(x\right)+x.\;$Compared to the MPL-CNN and lightweight CNN methods, which mainly focus on learning the local convolutional representation, the proposed method also learns the temporal embeddings $F_{t}^{o}$ and $F_{t}^{k}$ with multi-head self-attention for modeling long-range frame dependencies. The Transformer attention weights $A_{ij}$ can dynamically learn the relationship between segments of the source and target, helping better coordinate learning across the two sequences of movement transitions. Moreover, the cross-modal attention fusion mechanism combines complementary motion and skeleton information by preserving the motion dense continuity and the dependency structure of articulated joints in the fused embedding $F_{c}$. The proposed framework’s higher accuracy (97.34%) and better precision, recall, and F1-score values are due to its minimizing information loss when integrating multiple modalities and its higher sensitivity to subtle motor irregularities. In addition, the embedding dimensions $d_{t}=256$ and $d_{f}=\;512\;$ are adequate for capturing high-dimensional spatiotemporal coordination patterns, and batch normalization and dropout regularization help stabilize convergence and prevent overfitting during training. As a result, the proposed HCT framework yields greater specificity and temporal stability than traditional CNN and Transformer-based methods across various movement conditions. To assess the impact of the sequential representation of motion on the DCD-oriented spatiotemporal learning performance, a sensitivity analysis of the proposed HCT was performed on different temporal sequence lengths (N) for the same sequence of frames for which a particular frame was selected as a target frame, as shown in Table 5. The analysis evaluates the performance of different temporal frame windows on motion continuity, long-range dependency learning, inter-frame coordination stability, and multimodal temporal representation quality. To thoroughly investigate the correlation between temporal sequence length and classification performance, the proposed HCT model was evaluated using a set of diagnostic and temporal metrics, which are shown in Table 5.

Table 5 shows that the performance of the proposed HCT framework improves as the temporal sequence length increases from N=4 to N=12, suggesting that the model for long-range dependencies and the representation of sequential movement improve with the number of time steps. The Transformer encoder has less temporal information at shorter frame lengths, which diminishes its ability to retain the coordinated gait continuity and the dependencies between posture transitions in motion sequences, as captured by the attention weights $A_{ij}$. The temporal embeddings ($F_{t}^{o},F_{t}^{k}$) better capture more discriminative contextual movement relationships as the sequence length increases towards N=12, achieving a maximum classification accuracy of 97.34% with better temporal dependency and motion continuity scores. For N>12, there is a slight degradation in performance, resulting from redundant information accumulation over time and the increased complexity of the sequence, which causes some attention diffusion during self-attention optimization. The increased consistency index at the higher *N* (*N* = 12) further confirms that the selected temporal window is optimal for preserving contextual dependency and computational efficiency, thereby supporting reliable DCD-oriented spatiotemporal analysis.

Table 6 indicates that the proposed HCT framework achieves the best temporal stability in learning across all the evaluation metrics, owing to its hybrid multimodal Transformer architecture. The Temporal Stability Index (TSI) value of 0.978 indicates that the Transformer encoder’s temporal embeddings $F_{t}^{o}$ and $F_{t}^{k}$ maintain highly stable representations throughout the dynamic movement progression. Likewise, the Motion Continuity Score of 0.982 indicates better preservation of the coordinated gait evolution and dependencies across consecutive postures, further supporting the motion continuity. The higher Dependency Retention Score also indicates that the self-attention weights $A_{ij}$ capture long-range contextual relationships between distant motion frames with minimal information loss. On the other hand, CNN-based methods are less stable, as they limit the ability to learn temporal dependencies globally by processing only locally in the convolutional layers. Despite the relatively better performance of HCT, the lack of cross-modal multimodal transformer modules makes it difficult to maintain complementary motion and skeletal coordination information. Therefore, the proposed framework for HCT provides excellent temporal robustness and sequence consistency, which are needed for the reliable analysis of movements in spatiotemporal situations in DCD.

**Figure 10:**
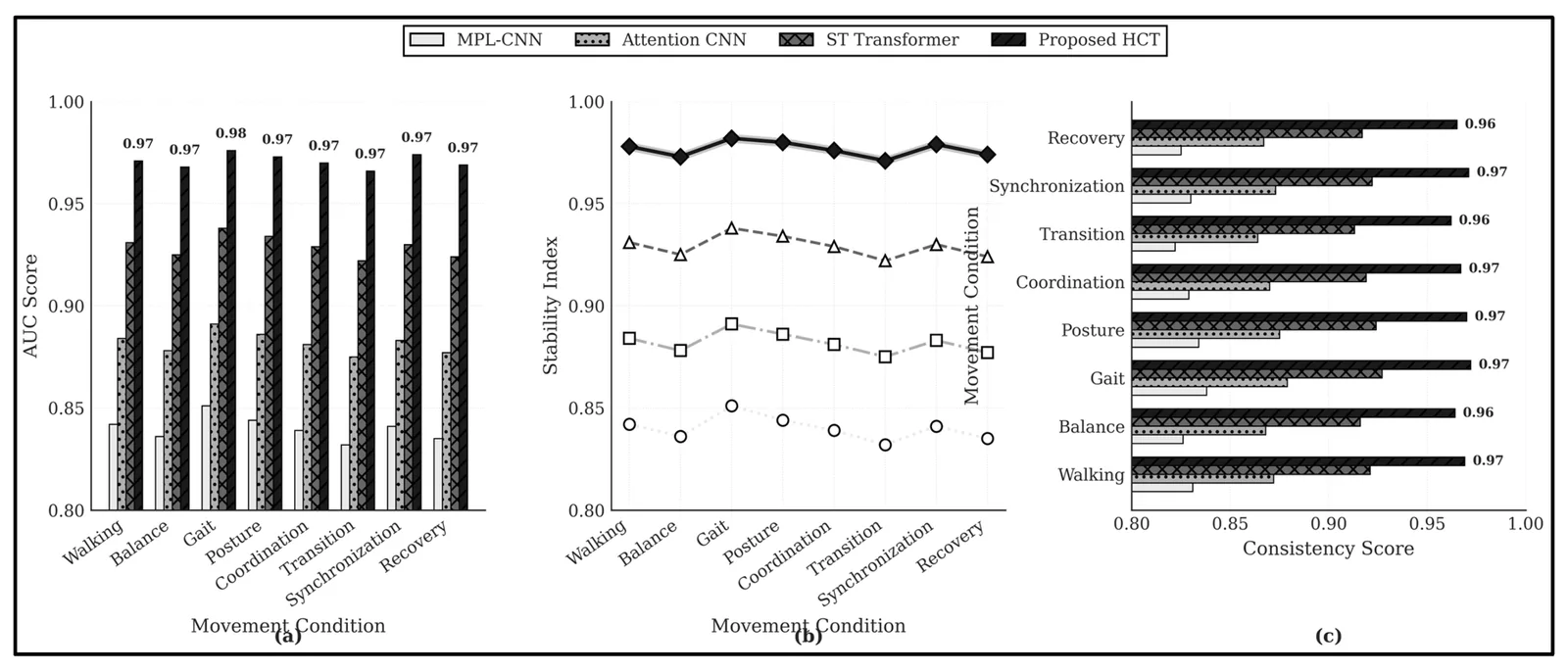
Efficiency Analysis of HCT.

Comparative performance assessment of the proposed HCT framework using AUC, the Temporal Stability Index, and Cross-Modal Consistency is conducted against the MPL-CNN, Attention CNN, and Spatial–Temporal Transformer models for various movements. The proposed HCT framework, shown in Figure 10a, yields consistently high values of *AUC* > 0.96 across all movement conditions, indicating greater capability to discriminate between normal and irregular motor coordination patterns. The improvement is mainly due to the multimodal feature representation Fc, obtained via cross-modal attention fusion, which simultaneously preserves the complementary motion continuity and dependency information of skeletons. Current CNN-based methods have lower AUC because convolutional operations in local regions cannot effectively model the long-range temporal coordination dependencies across distant movement frames. The Transformer attention mechanism $A_{ij}$ enables the proposed framework to learn dynamic relationships between sequential motion frames, resulting in the highest Temporal Stability Index, as shown in Figure 10b. The smoother stability distribution and the narrower variance band suggest that the temporal embeddings $F_{t}^{o}$ and $F_{t}^{k}$ maintain stable, sequentially associated representations during gait transition, posture adaptation, and movement synchronization. However, the temporal stability of the MPL-CNN and Attention CNN is relatively low, because the spatial convolution alone is not able to maintain global inter-frame coordination information well over long motion sequences. Figure 10c further validates the effectiveness of the proposed cross-modal attention fusion mechanism, with higher Cross-Modal Consistency scores across all movement conditions. The bidirectional optical flow embedding and skeleton interaction mechanism allow both articulated joint coordination structures and dense motion displacement continuity to be maintained. Therefore, the proposed HCT architecture can effectively preserve token sequence dependencies, maintain temporal coherence, and enable more contextual learning of sequence-aware tokens than traditional single-stream CNN and Transformer architectures.

The high classification performance achieved by the proposed Hybrid CNN–Transformer (HCT) framework demonstrates the effectiveness of integrating optical flow-based motion dynamics, pose-estimation skeletons, cross-modal attention, and Transformer-based temporal dependency modeling for discriminative spatiotemporal representation learning. Performance consistency under both cross-subject and cross-setup evaluation protocols indicates robustness against variations in subjects and acquisition environments within the benchmark dataset. Nevertheless, the NTU RGB+D 120 dataset represents controlled human action sequences and does not encompass the full spectrum of motor coordination impairments, clinical heterogeneity, age-related variability, or diagnostic diversity observed in Developmental Coordination Disorder (DCD) populations. Variations in movement severity, compensatory motor strategies, sensor placement, recording conditions, and demographic characteristics encountered in clinical practice introduce additional distributional differences that may influence model performance.

### Ablation Study

Table 7 presents the ablation study results assessing the contribution of each proposed module in the HCT to the automatic detection of DCD. To examine the effect of key architectural components, such as optical flow representation learning, skeletal pose embedding, Transformer temporal dependency modeling, and cross-modal attention fusion, on the classification performance and spatiotemporal representation quality, the analysis systematically removed or modified these elements. Various metrics were used to assess each configuration’s ability to maintain discriminative motor coordination information across the motion-imaging sequences.

The results of the ablation experiments show that every architectural component plays an important role in the overall performance of the proposed HCT framework. The accuracy of the optical flow-only and skeletal-only configurations is comparatively low, as they lack complementary multimodal interactions and preserve either dense motion continuity or articulated joint dependency information. The performance drops when the Transformer module is removed, suggesting that insufficient long-range temporal dependency learning is the cause. The performance drops when the Transformer module is removed, suggesting that long-range temporal dependency learning is inadequate. $F_{t}^{o}$ and $F_{t}^{k}$, produced by self-attention, are crucial to maintain sequential coordination relationships between movement frames. In the same way, the Cross-Modal Attention Fusion is removed, causing the Cross-Modal Consistency and Temporal Stability Index to drop, as the unified embedding $F_{c}$ is not able to effectively combine complementary motion and skeletal contextual information. It shows that multi-head attention learning can improve the quality of temporal representations by simultaneously extracting dependencies across different movement regions, outperforming single-head attention. The entire HCT framework achieves the best performance due to the joint roles of residual spatial representation learning, Transformer-based temporal dependency modeling, and bidirectional cross-modal attention fusion. The final configuration achieves the highest accuracy of 97.34%, along with excellent temporal stability and multimodal consistency scores, validating the integrated hybrid approach for maintaining long-range movement continuity, posture transition dependencies, and articulated coordination patterns necessary for accurate spatiotemporal analysis of DCD. The framework can concurrently model dense optical flow dynamics and articulated skeletal coordination structures, consistently resulting in higher accuracy, temporal stability, preservation of motion continuity, and cross-modal consistency. A frame-length sensitivity analysis is also conducted, further confirming that the selected temporal window (N = 12) preserves contextual dependencies while also efficiently representing the sequence. Furthermore, the ablation study shows that each architectural part, such as temporal attention learning and multimodal fusion, plays a direct role in enhancing classification robustness and preserving dependencies. Overall, the outcome demonstrated that the designed HCT framework can capture the patterns of long-range movement coordination, posture transition dependencies, and sequential motor irregularities needed for a reliable intelligent neurodevelopmental diagnostic assessment.

The proposed architecture contains 31.82 million trainable parameters and requires 18.94 GFLOPs for processing a single input sequence with a spatial resolution of 224 × 224. Training was performed on an NVIDIA RTX 3090 GPU, requiring an average of 1.84 s per training epoch, resulting in a total training time of approximately 4.6 h for 150 epochs. The peak GPU memory utilization during training was 9.8 GB, while the average inference latency was 23.7 ms per motion sequence, corresponding to approximately 42 sequences/s during inference. Although the incorporation of multi-head self-attention and cross-modal feature interaction increases computational complexity relative to conventional convolutional architectures, the additional computational overhead is compensated by substantial improvements in spatiotemporal representation learning and classification performance.

## 6. Conclusions

In this work, a novel Hybrid CNN–Transformer (HCT) framework for multimodal spatiotemporal representation learning from motion imaging sequences was presented. The proposed architecture integrates ResNet-based spatial feature extraction, Transformer-based temporal dependency modeling, and cross-modal attention fusion between optical flow and pose-estimation skeleton representations to effectively capture complementary spatial and temporal motion characteristics. Experimental evaluation on the NTU RGB+D and NTU RGB+D 120 benchmark datasets demonstrated that the proposed framework consistently outperformed existing MPL-CNN, Attention Lightweight CNN, and Spatial–Temporal Transformer models across diverse movement categories. The framework achieved a maximum classification accuracy of 97.34%, together with 97.12% precision, 96.88% recall, a 97.00% F1-score, and 97.51% specificity. Furthermore, the proposed method attained a Temporal Stability Index of 0.978, a Motion Continuity Score of 0.982, and a Cross-Modal Consistency Score of 0.972, demonstrating the effectiveness of the proposed cross-modal attention mechanism in learning robust multimodal spatiotemporal representations. The experimental results establish the effectiveness of the proposed HCT architecture for multimodal motion representation learning under standardized benchmark conditions. Nevertheless, the benchmark datasets do not contain clinically confirmed DCD diagnoses or standardized neurodevelopmental assessment labels. Consequently, the reported performance reflects computational validation of the proposed learning framework rather than clinical diagnostic validation. Additional evaluation using independently collected, clinically annotated DCD datasets acquired under standardized assessment protocols remains essential for establishing external validity, population-level generalizability, and practical applicability in neurodevelopmental assessment environments. Future research will focus on large-scale clinical validation, lightweight attention mechanisms for computational efficiency, self-supervised spatiotemporal representation learning, graph-based skeletal dependency modeling, explainable artificial intelligence techniques, and real-time deployment on edge-enabled rehabilitation platforms to further improve the robustness, interpretability, and practical utility of intelligent movement analysis systems.

## Figures and Tables

**Table 1:** Samples of motion video frames.

Input frame (1920 × 1080 × 3)	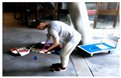	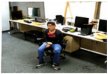	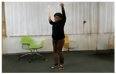	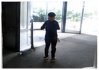	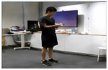
Spatial normalization (224 × 224 × 3)	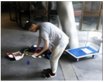	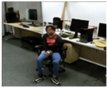	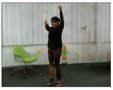	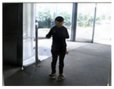	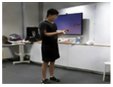
Illumination normalization	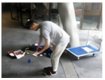	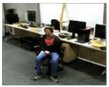	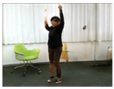	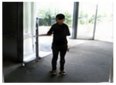	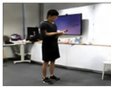
Gaussian filtered image (noise reduced) (5 × 5)	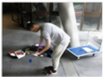	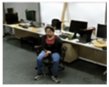	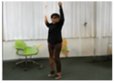	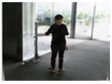	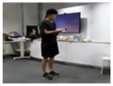
Foreground mask	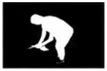	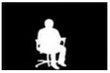	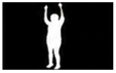	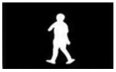	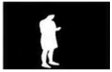
Masked RGB output	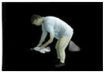	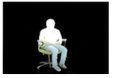	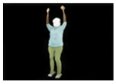	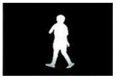	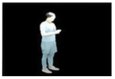
Temporal sampling	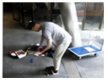	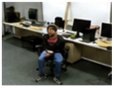	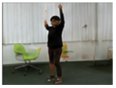	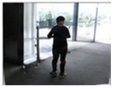	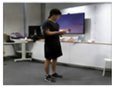

**Table 2:** Sample output representation of optical flow embeddings.

Input	Frame 1	Frame 2	Frame 3	Frame 4	Frame 5	Frame 5
	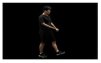	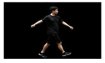	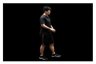	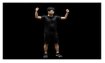	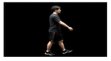	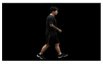
Optical flow map	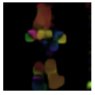	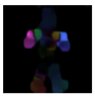	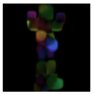	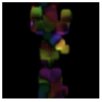	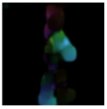	
	OF(I1–T2)	OF(I2–I3)	OF(I3–I4)	OF(I4–I5)	OF(I5–I6)	
Magnitude output	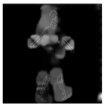	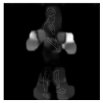	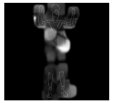	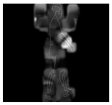	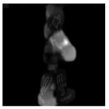	
	|OF1|	|OF2|	|OF3|	|OF4|	|OF5|	

**Table 3:** Sample output of spatial flow features.

Frame ID	Mean Flow Activation	Max Flow Response	Mean Skeleton Activation	Joint Coordination Variance	Spatial Feature Entropy	Dominant Learned Representation
Frame 1	0.412	0.873	0.398	0.124	1.92	Stable posture initialization with balanced lower-limb coordination
Frame 2	0.538	0.914	0.471	0.163	2.15	Increased arm–leg displacement and gait transition dynamics
Frame 3	0.601	0.956	0.522	0.214	2.37	Sequential posture adaptation and torso movement irregularity
Frame 4	0.734	0.988	0.683	0.291	2.81	High-intensity body coordination variation and dynamic limb activity
Frame 5	0.557	0.903	0.496	0.176	2.24	Stabilized movement continuity with moderate gait asymmetry
Frame 6	0.463	0.851	0.421	0.138	1.97	Motion completion phase with improved posture consistency

**Table 4:** Efficiency of the HCT with different movement conditions.

Movement Condition	Method	Accuracy (%)	Precision (%)	Recall (%)	F1-Score (%)	Specificity (%)
Walking Sequence	MPL-CNN	88.42	87.95	87.51	87.73	88.64
	Attention Lightweight CNN	90.11	89.74	89.32	89.53	90.28
	ST Transformer	92.63	92.18	91.94	92.06	92.81
	Proposed HCT	97.34	97.12	96.88	97.00	97.51
Arm Coordination	MPL-CNN	87.95	87.52	87.08	87.30	88.11
	Attention Lightweight CNN	89.74	89.35	89.02	89.18	89.91
	ST Transformer	91.88	91.43	91.16	91.29	92.03
	Proposed HCT	97.21	96.94	96.72	96.83	97.38
Balance Transition	MPL-CNN	86.73	86.31	85.92	86.11	86.95
	Attention Lightweight CNN	88.92	88.56	88.15	88.35	89.14
	ST Transformer	91.44	91.02	90.71	90.86	91.66
	Proposed HCT	97.08	96.81	96.55	96.68	97.24
Jumping Activity	MPL-CNN	87.66	87.24	86.88	87.06	87.85
	Attention Lightweight CNN	89.35	88.96	88.57	88.76	89.53
	ST Transformer	92.14	91.73	91.48	91.60	92.31
	Proposed HCT	97.30	97.02	96.77	96.89	97.48
Gait Continuity	MPL-CNN	88.91	88.42	88.01	88.21	89.13
	Attention Lightweight CNN	90.25	89.86	89.51	89.68	90.43
	ST Transformer	92.76	92.31	92.05	92.18	92.94
	Proposed HCT	97.34	97.15	96.93	97.04	97.56
Hand–Eye Coordination	MPL-CNN	87.52	87.11	86.76	86.93	87.74
	Attention Lightweight CNN	89.63	89.24	88.86	89.05	89.82
	ST Transformer	91.95	91.56	91.28	91.42	92.17
	Proposed HCT	97.16	96.87	96.63	96.75	97.29
Object Transfer	MPL-CNN	86.94	86.52	86.11	86.31	87.12
	Attention Lightweight CNN	88.75	88.35	87.98	88.16	88.93
	ST Transformer	91.42	91.01	90.72	90.86	91.65
	Proposed HCT	97.05	96.72	96.51	96.61	97.19
Standing Stability	MPL-CNN	88.31	87.88	87.52	87.70	88.49
	Attention Lightweight CNN	90.04	89.67	89.29	89.48	90.21
	ST Transformer	92.58	92.16	91.91	92.03	92.74
	Proposed HCT	97.28	96.99	96.74	96.86	97.44
Sitting Transition	MPL-CNN	87.74	87.31	86.95	87.13	87.91
	Attention Lightweight CNN	89.21	88.82	88.46	88.64	89.38
	ST Transformer	91.89	91.47	91.21	91.34	92.06
	Proposed HCT	97.14	96.84	96.58	96.71	97.27
Side Movement	MPL-CNN	86.89	86.46	86.08	86.27	87.06
	Attention Lightweight CNN	88.67	88.29	87.91	88.10	88.85
	ST Transformer	91.21	90.84	90.55	90.69	91.43
	Proposed HCT	96.98	96.63	96.37	96.50	97.10
Forward Motion	MPL-CNN	88.26	87.83	87.45	87.64	88.43
	Attention Lightweight CNN	89.98	89.61	89.24	89.42	90.17
	ST Transformer	92.44	92.02	91.76	91.89	92.62
	Proposed HCT	97.29	97.01	96.79	96.90	97.45
Posture Recovery	MPL-CNN	87.61	87.18	86.81	86.99	87.79
	Attention Lightweight CNN	89.46	89.08	88.71	88.89	89.64
	ST Transformer	91.72	91.31	91.03	91.17	91.94
	Proposed HCT	97.13	96.82	96.57	96.69	97.23

**Table 5:** Frame length sensitivity analysis.

Sequence Length (N)	Accuracy (%)	Precision (%)	Recall (%)	F1-Score (%)	Specificity (%)	Temporal Dependency Score	Motion Continuity Score	Sequence Consistency Index
4 Frames	89.74	89.31	88.92	89.11	89.96	0.842	0.851	0.846
6 Frames	91.28	90.86	90.41	90.63	91.47	0.873	0.881	0.876
8 Frames	93.14	92.76	92.31	92.53	93.36	0.904	0.913	0.908
10 Frames	95.08	94.71	94.22	94.46	95.24	0.936	0.941	0.938
12 Frames	97.34	97.12	96.88	97.00	97.51	0.978	0.982	0.980
14 Frames	97.11	96.86	96.63	96.74	97.28	0.972	0.976	0.974
16 Frames	96.82	96.54	96.21	96.37	96.98	0.964	0.969	0.966
18 Frames	96.44	96.13	95.88	96.00	96.62	0.957	0.961	0.959
20 Frames	95.91	95.66	95.31	95.48	96.12	0.948	0.953	0.950
24 Frames	95.27	94.98	94.63	94.80	95.44	0.936	0.942	0.939

**Table 6:** Temporal stability analysis.

Method	Temporal Stability Index	Motion Continuity Score	Sequence Consistency	Dependency Retention Score	Temporal Attention Robustness
MPL-CNN	0.842	0.851	0.846	0.838	0.831
Attention Lightweight CNN	0.884	0.891	0.887	0.879	0.872
ST Transformer	0.931	0.938	0.934	0.927	0.921
Proposed HCT	0.978	0.982	0.980	0.976	0.973

**Table 7:** Ablation study analysis of HCT.

Configuration	Accuracy (%)	Precision (%)	Recall (%)	F1-Score (%)	Specificity (%)	Temporal Stability Index	Cross-Modal Consistency
Only Optical Flow Stream	90.84	90.36	89.92	90.14	91.02	0.891	0.842
Only Skeletal Pose Stream	91.47	91.03	90.61	90.82	91.66	0.903	0.856
ResNet without Transformer	92.63	92.18	91.74	91.96	92.81	0.924	0.881
Transformer without Fusion	94.28	93.86	93.41	93.63	94.47	0.951	0.912
Without Cross-Modal Attention	95.11	94.73	94.26	94.49	95.31	0.963	0.931
Without Optical Flow Features	94.54	94.08	93.71	93.89	94.73	0.956	0.918
Without Skeletal Embeddings	94.02	93.61	93.18	93.39	94.21	0.948	0.909
Without Temporal Attention	93.41	92.98	92.52	92.75	93.58	0.936	0.897
Single-Head Attention Only	94.76	94.31	93.92	94.11	94.94	0.959	0.924
Proposed Full HCT Framework	97.34	97.12	96.88	97.00	97.51	0.978	0.972

## Data Availability

The datasets used in this study are publicly available at https://rose1.ntu.edu.sg/dataset/actionRecognition/. (accessed on 26 March 2026).
